# Comparative Effectiveness of Different Exercises for Reducing Pain Intensity in Primary Dysmenorrhea: A Systematic Review and Network Meta-analysis of Randomized Controlled Trials

**DOI:** 10.1186/s40798-024-00718-4

**Published:** 2024-05-30

**Authors:** I-Chen Tsai, Chih-Wei Hsu, Chun-Hung Chang, Wei-Te Lei, Ping-Tao Tseng, Ke-Vin Chang

**Affiliations:** 1 Institute of Clinical Medicine, National Yang Ming Chiao Tung University, Taipei, Taiwan; 2 Congenital Heart Disease Study Group, Asian Society of Cardiovascular Imaging, Seoul, Korea; 3 InnovaRad Inc., Taichung, Taiwan; 4 Department of Psychiatry, Kaohsiung Chang Gung Memorial Hospital and Chang Gung University College of Medicine, Kaohsiung, Taiwan; 5 Department of Computer Science and Information Engineering, National Cheng Kung University, Tainan, Taiwan; 6 Institute of Clinical Medical Science, China Medical University, Taichung, Taiwan; 7 Department of Psychiatry and Brain Disease Research Center, China Medical University Hospital, Taichung, Taiwan; 8 An Nan Hospital, China Medical University, Tainan, Taiwan; 9 Section of Immunology, Rheumatology, and Allergy Department of Pediatrics, Hsinchu Mackay Memorial Hospital, Hsinchu, Taiwan; 10 Graduate Institute of Clinical Medical Sciences, College of Medicine, Chang Gung University, Taoyuan, Taiwan; 11 Prospect Clinic for Otorhinolaryngology and Neurology, Kaohsiung, Taiwan; 12 Institute of Biomedical Sciences, National Sun Yat-Sen University, Kaohsiung, Taiwan; 13 Department of Psychology, College of Medical and Health Science, Asia University, Taichung, Taiwan; 14 Institute of Precision Medicine, National Sun Yat-Sen University, Kaohsiung City, Taiwan; 15 Department of Physical Medicine and Rehabilitation, National Taiwan University Hospital and National Taiwan University College of Medicine, No. 1, Changde St., Zhongzheng Dist., Taipei City, 100229 Taiwan; 16 Department of Physical Medicine and Rehabilitation, National Taiwan University Hospital, Bei-Hu Branch, Taipei, Taiwan; 17 Center for Regional Anesthesia and Pain Medicine, Taipei Medical University, Wang-Fang Hospital, Taipei, Taiwan

## Abstract

**Background:**

Studies have demonstrated that exercise can mitigate the intensity of menstrual pain in primary dysmenorrhea, but the most effective type of exercise remains unclear. The objective of this systematic review and network meta-analysis was to evaluate the effectiveness of different exercise regimens in reducing pain associated with primary dysmenorrhoea.

**Methods:**

Randomized controlled trials investigating the relationship between menstrual pain and exercise were selected from major electronic databases until February 2, 2024. The primary outcome was the effect of exercise on pain intensity measured by the mean difference on a 10-cm visual analogue scale at 4 and 8 weeks after intervention. The secondary outcome was the difference in risk of dropout at 8 weeks. The study protocol was registered as INPLASY202330050.

**Results:**

This systematic review and network meta-analysis included 29 randomized controlled trials, which involved 1808 participants with primary dysmenorrhea. Exercise interventions included relaxation exercise, strength training, aerobic activity, yoga, mixed exercise, and the Kegel maneuver. Relaxation exercise was the most effective in reducing menstrual pain in 4 weeks (− 3.56; 95% confidence interval: − 5.03 to − 2.08). All exercise interventions were effective in reducing menstrual pain at 8 weeks, with reductions ranging from − 3.87 (95% CI − 5.51 to − 2.22) for relaxation exercise to − 2.75 (95% CI − 4.00 to − 1.51) for yoga, compared to the control group. Relaxation exercises were found to have a significantly lower dropout risk (− 0.11; 95% CI  − 0.20 to 0.02), while none of the exercise types was associated with a higher dropout risk than the control group.

**Conclusion:**

All exercise interventions were effective in reducing menstrual pain in primary dysmenorrhea after 8 weeks of intervention. However, relaxation exercise was found to be the most effective intervention at 4 and 8 weeks and had the lowest risk of dropout.

**Graphical Abstract:**

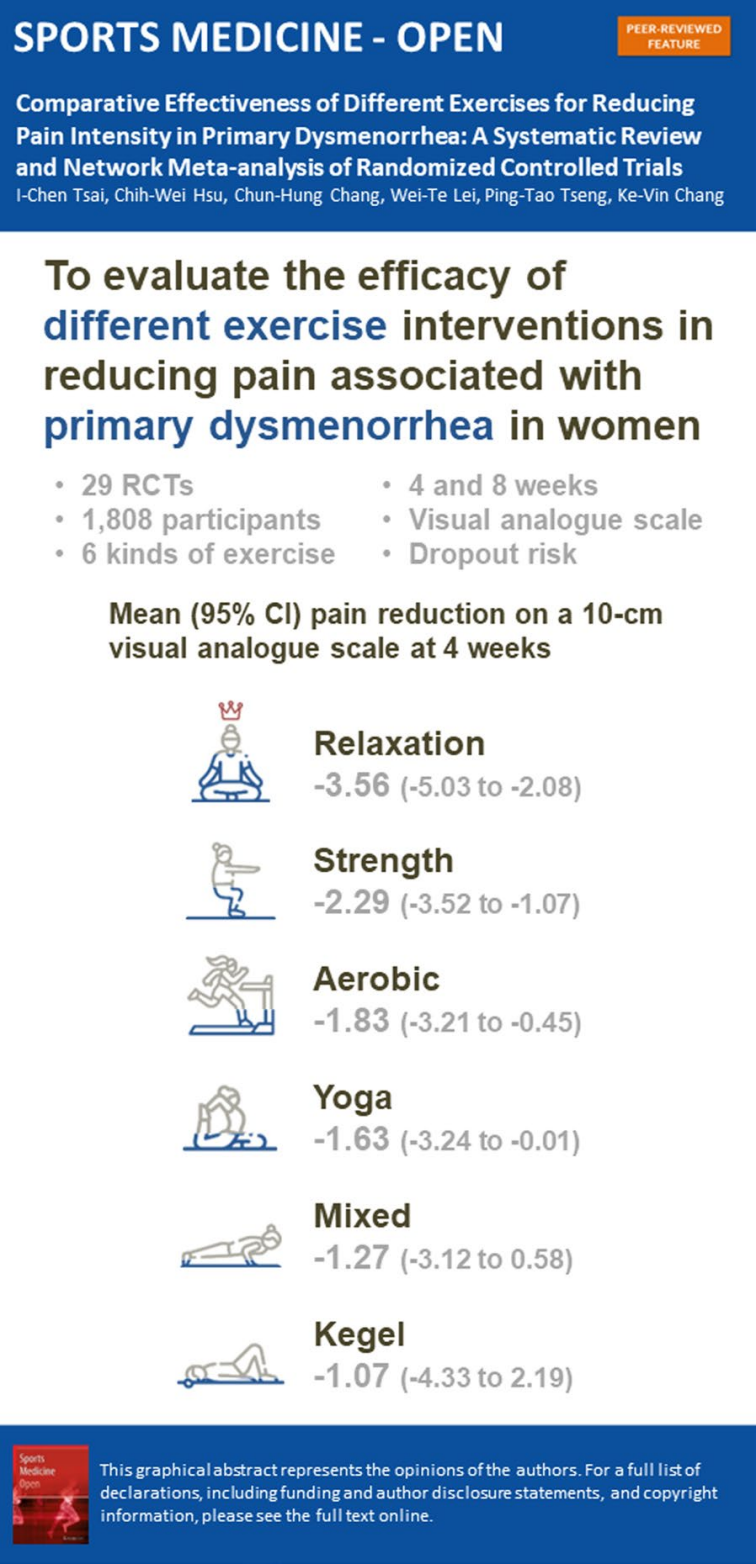

**Supplementary Information:**

The online version contains supplementary material available at 10.1186/s40798-024-00718-4.

## Plain Language Summary

This analysis aimed to see how effective different types of exercise are in reducing pain in women with primary dysmenorrhea. The researchers looked at 29 studies involving 1808 participants and evaluated six different types of exercise. The main outcome was the effect of pain reduction after 4 and 8 weeks of exercise. The researchers found that all types of exercise were effective in reducing menstrual pain after 8 weeks, with relaxation exercises being the most effective at both 4 and 8 weeks. None of the exercise types were associated with higher dropout risks than the control group, and relaxation exercise had a lower dropout risk than the other types of exercise.

**Supplementary Information:**

The online version contains supplementary material available at 10.1186/s40798-024-00718-4.

## Key Points

1. Exercise can be an effective way to reduce menstrual pain in women with primary dysmenorrhea.
2. Different types of exercise, including relaxation exercise, strength training, aerobic activity, yoga, mixed exercise, and the Kegel maneuver, have been found to be effective in reducing menstrual pain after 8 weeks.
3. Relaxation exercises, such as progressive muscle relaxation and self-administered massage, have been found to be the most effective interventions for reducing menstrual pain at both 4 and 8 weeks, and they also have a lower risk of dropout.

**Supplementary Information:**

The online version contains supplementary material available at 10.1186/s40798-024-00718-4.

## Introduction

Primary dysmenorrhea, prevalent among women of reproductive age, manifests as menstrual pain and discomfort localized in the lower abdomen. Globally, this condition afflicts a substantial proportion of women, with reported prevalence rates varying from 17 to 90% [1]. Notably, primary dysmenorrhea exerts a considerable impact on the quality of life, often resulting in absenteeism from occupational or educational settings, diminished productivity, and escalated healthcare demands. In the United States alone, the economic burden attributed to this condition is estimated at approximately 600 million working hours lost per year, equating to a financial loss of around US$2 billion annually [2].

Primary dysmenorrhea is postulated to arise from the synthesis of prostaglandins [1, 3] during menstruation, which precipitates potent uterine contractions, diminished uterine blood flow, and subsequent activation of nociceptors. While non-steroidal anti-inflammatory drugs represent the principal therapeutic approach for this condition [1, 3], their efficacy in providing comprehensive relief is not universal, and prolonged usage is associated with potential adverse effects. Consequently, there is an increasing interest in non-pharmacological modalities, such as physical exercise, as potential adjunctive treatments for primary dysmenorrhea.

Owing to the potential of exercise to augment endorphin secretion [4] and attenuate inflammation [5], a multitude of studies have scrutinized its efficacy in the management of primary dysmenorrhea [6–12]. Despite this extensive investigation, the scholarly discourse remains divided regarding the most effective form of exercise [13, 14]. Various modalities have been examined, encompassing relaxation exercises such as progressive muscle relaxation [12, 15] and self-administered massage [16, 17], alongside strength training [18], aerobic exercises [19], yoga [20], mixed exercise regimes [21], and the Kegel maneuver [22]. Certain studies have documented substantial alleviation in pain following only a 4-week period of exercise intervention [15], whereas others indicate a necessity for up to 8 weeks to discern any tangible benefits [11]. Furthermore, the incidence of participant withdrawal has exhibited variability across these diverse exercise protocols [12, 23].

In contrast to randomized controlled trials and conventional pairwise meta-analyses [24, 25], a meticulously conducted network meta-analysis offers expanded insights into the comparative efficacy and acceptability of various treatments for a specified clinical condition [26]. Moreover, this type of analysis is capable of evaluating the potential superiority of individual interventions in achieving specific outcomes. Such information is pivotal for the formulation of evidence-based clinical guidelines and plays a critical role in informing the design and focus of future clinical research [27, 28].

The objective of this network meta-analysis was to systematically rank and compare the mean differences in pain reduction and the differences in risk of dropout associated with diverse exercise interventions in patients with primary dysmenorrhea. The collation and analysis of these data are intended to furnish more precise and tailored recommendations for individuals contemplating exercise as a strategy for pain management in the context of primary dysmenorrhea.

## Methods

This study was conducted adhering to the Preferred Reporting Items for Systematic Reviews and Meta-Analyses (PRISMA) extension for network meta-analysis (PRISMA NMA) guidelines [29] (Additional file 1: Table S1). Registration of the study was completed with INPLASY, bearing the registration number INPLASY202330050 [30]. Given the nature of this research, the requirement for ethics review board approval and participant informed consent was deemed unnecessary.

### Database Searches and Study Identification

The literature search for this systematic review and network meta-analysis was independently conducted by two authors (Tsai IC and Lei WT). They utilized electronic databases including PubMed, Cochrane Reviews, Cochrane CENTRAL, Web of Science, and ClinicalTrials.gov. The search was structured around a series of keywords: (‘primary dysmenorrhea’ OR ‘dysmenorrhea’ OR ‘menstrual cramps’ OR ‘painful periods’) AND (‘exercise’ OR ‘yoga’ OR ‘aerobic’ OR ‘training’ OR ‘sports’ OR ‘physical activity’ OR ‘workout’ OR ‘fitness’ OR ‘training’) AND (‘randomized’ OR ‘randomised’ OR ‘random’). This search strategy, detailed in Additional file 1: Table S2 of the Supplementary Material, encompassed records from the inception of each database until the final search date of February 2, 2024.

During the preliminary phase of this research, two authors were assigned the responsibility of assessing the relevance of the titles and abstracts of studies identified in the search, employing a consensus-based approach for determining their eligibility. This evaluation was performed using the previously mentioned databases to meticulously review potential trials. In addition to electronic searches, the reference lists of several review articles [13, 14, 31–36] were also meticulously perused, and manual searches were conducted to ensure comprehensive coverage. In instances where the initial two reviewers encountered difficulties in achieving a consensus, a third reviewer and co-author of the study (Chang KV) was engaged for arbitration. It is noteworthy that this search process did not impose any language restrictions.

### Inclusion and Exclusion Criteria

The network meta-analysis adopted the PICO framework (population, intervention, comparison, outcome) with the following specifications: (1) P: female human participants with primary dysmenorrhea; (2) I: exercise interventions; (3) C: control group without intervention; and (4) O: changes in pain intensity. The diagnosis of primary dysmenorrhea can be established through adherence to the criteria prescribed by the American College of Obstetricians and Gynecologists, as per their recommended guidelines [37].

The study employed the following inclusion criteria: (1) randomized controlled trials that recruited female human participants with primary dysmenorrhea, (2) randomized controlled trials that investigated the quantitative assessment of pain intensity pre- and post-exercise, (3) the control group received no intervention or regular care, and (4) trials that had available data on pain intensity assessment pre- and post-intervention at either 4 weeks or 8 weeks [12, 20, 21, 38].

The selection of the 4-week and 8-week evaluation time points was based on an early stage exploratory literature review. This review revealed that these durations were the most frequently utilized assessment intervals in relevant studies [10–12, 16, 18, 20–23, 38–40], typically aligning with the completion of the first or second menstrual cycle post-intervention. By examining outcomes at these two distinct intervals, our analysis aimed to elucidate the requisite duration of each exercise intervention necessary to exert a significant impact on the pain intensity associated with primary dysmenorrhea.

Exclusion criteria for this review and network meta-analysis included: (1) non-randomized controlled trials, (2) studies without comparisons of exercise vs. exercise or exercise vs. regular care comparison, (3) studies lacking quantitative assessments of pain intensity, (4) pain intensity assessment methods that could not be linearly converted to a 10 cm visual analogue scale (VAS), since the VAS is the most convenient method for quantifying pain intensity [41] and is widely used in studies related to primary dysmenorrhea [23, 39, 42], (5) studies that did not separately evaluate pain intensity, but were included this with other attributes such as distress or duration, (6) studies with incomplete or unavailable data, even after attempts to contact the authors via email, and (7) studies enrolling participants that overlapped with a published trial already enrolled in our analysis.

### Modeling for Network Meta-analysis

In the execution of this network meta-analysis, specific methodological principles were rigorously observed during the model’s construction. To mitigate the risk of excessive heterogeneity within the analysis, the scope of paired comparisons was deliberately confined to either exercise versus exercise or exercise versus regular care modalities. Consequently, comparisons involving exercise and other treatment options, such as non-steroidal anti-inflammatory drugs, transcutaneous electrical nerve stimulation, or various nutritional supplements, were systematically excluded. The rationale for this exclusion was predicated on the premise that the incorporation of a broader array of treatments could potentially introduce divergent network geometries. This variability, attributable to the differences in the nature of the therapies under consideration, could potentially precipitate inconsistent outcomes within the network meta-analysis [43].

In our study, the categorization of exercise types was meticulously conducted based on the specific content of the exercise prescription [44]. This process entailed a detailed discussion and analysis by two authors (Tsai IC and Lei WT), followed by a validation from a rehabilitation physician (Chang KV). For example, exercises that are conventionally recognized as strength training, such as squats and heel raises, were classified under the strength training category. This classification was upheld even in cases where the original authors of the studies might have referred to these exercises as stretching exercises [42, 45, 46]. The decision to categorize exercises in this manner was grounded in the actual exercise regimen prescribed in the studies, rather than solely on the terminology used by the original study authors.

### Methodological Quality Appraisal

In order to evaluate the methodological quality of the studies incorporated into our analysis, we utilized the Cochrane risk of bias tool for randomized trials (RoB 2, version 2, based in London, United Kingdom) [47]. This tool is designed with six fundamental domains that are critical for assessing the integrity and rigor of a study. These domains encompass the randomization process, adherence to the intervention protocol, management of missing outcome data, accuracy of outcome measurement, potential for selective reporting, and the overall assessment of bias risk. Each of these elements plays a pivotal role in determining the reliability and validity of the findings reported in the randomized trials under review.

### Primary Outcome: Pain Intensity Reduction, Mean Difference

In this study, the primary outcomes assessed were the changes in pain intensity, quantified using the VAS, subsequent to exercise intervention or regular care. We incorporated data from evaluations conducted at both the 4-week and 8-week time points for comprehensive analysis.

To quantify these primary study outcomes, specifically the changes in pain intensity, we employed the mean difference along with 95% confidence intervals (95% CI). This methodological choice was predicated on the aim of rendering the results more accessible and interpretable for readers. The use of mean difference values facilitates a straightforward visualization of the extent of change, expressed in terms of degrees on a 10 cm VAS scale dedicated to measuring pain intensity. Such an approach enhances the clarity and comprehensibility of the outcome measures, aiding in the practical application and understanding of the study’s findings.

### Secondary Outcome: Difference in Risk of Dropout

The secondary outcome measure in our study was the difference in risk of dropout at the 8th week, serving as an intuitive indicator of the sustainability and acceptability of the various exercise interventions. For example, consider a scenario where an individual opts for a particular exercise regimen to alleviate menstrual discomfort and encounters a dropout rate of 12%. In comparison, the control group, which does not receive any specific intervention and might subsequently seek medical assistance or initiate an exercise routine independently, shows a dropout rate of 7%. In this context, the difference in risk of dropout between the two groups would be 5%. This measure effectively reflects the relative adherence to the exercise regimen, offering insights into the feasibility and potential barriers to sustained participation in the prescribed exercise routines for managing primary dysmenorrhea.

### Data Extraction, Management and Conversion

The data extraction process from the evaluated studies was meticulously executed by two independent authors (Tsai IC and Lei WT). This comprehensive extraction encompassed various aspects such as demographic data, the study design, specific details of the exercise protocols implemented, and both primary and secondary outcome measures.

In instances where certain data elements were not directly accessible within the published articles, efforts were made to contact the corresponding authors of those studies to acquire the original data.

The process of data extraction, including the conversion and amalgamation of results from different study arms, was conducted in strict adherence to the guidelines outlined in the Cochrane Handbook for Systematic Reviews of Interventions, as well as recommendations from relevant medical literature [26, 48, 49]. This approach ensured a standardized, rigorous, and methodologically sound handling of the data, thereby enhancing the reliability and validity of the results derived from our network meta-analysis.

### Statistical Analyses

Due to the inclusion of multiple types of exercises, we employed a random-effects model for this network meta-analysis [50]. The analysis was conducted using MetaInsight (version 4.0.2, Complex Reviews Support Unit, National Institute for Health Research, United Kingdom) under a frequentist framework. MetaInsight is a web-based service for network meta-analysis with the statistical core based on R software, utilizing the *netmeta* package for frequentist statistical calculations [51].

Initially, a forest plot was generated to display all pairwise comparisons from all individual studies, along with a network plot. Subsequently, forest plots were created for mean differences in pain reduction at 4 and 8 weeks, as well as the differences in risks of dropout at 8 weeks, for each exercise type compared to the control group, to provide an overall summary of the effects. The effect sizes were presented as point estimates (95% CI). The exercise types were ranked, and the numerical values for both direct and indirect comparisons were presented in tables. Inconsistency tests were conducted to detect any data disparities. A two-tailed *p* value of less than 0.05 was considered statistically significant.

### Sensitivity Analysis

To convert baseline and post-intervention pain intensity measurements into mean changes and standard deviations, an assumption regarding the pre-post correlation coefficient is requisite. In this analysis, we adopted a coefficient of 0.8, aligning with the recommendations delineated in the Cochrane Handbook [52]. Notably, there exists a divergence of opinions among scholars regarding the optimal value of this coefficient, with commonly cited figures being 0.5, 0.7, and 0.8 [53].

To assess the potential impact of the selected coefficient on our study’s outcomes, we undertook a sensitivity analysis. This involved recalculating the effect sizes for changes in VAS values at both the 4-week and 8-week intervals, using a coefficient of 0.5 [53]. Subsequently, we scrutinized the direction and magnitude of the effects, their statistical significance, and the relative ranking of the results. This sensitivity analysis was crucial for ensuring the robustness of our findings and their independence from the specific assumption of the correlation coefficient.

### Publication Bias

Publication bias assessment adhered to the guidelines outlined in the Cochrane Handbook for Systematic Reviews of Interventions [26]. The generation of a funnel plot was performed concerning the comparison with the control group, utilizing Comprehensive Meta-Analysis software, version 4 (Biostat, Englewood, NJ). In cases where there were ten or more datasets available, an Egger’s regression test was employed.

## Results

### Study Identification and Selection

**Fig. 1:**
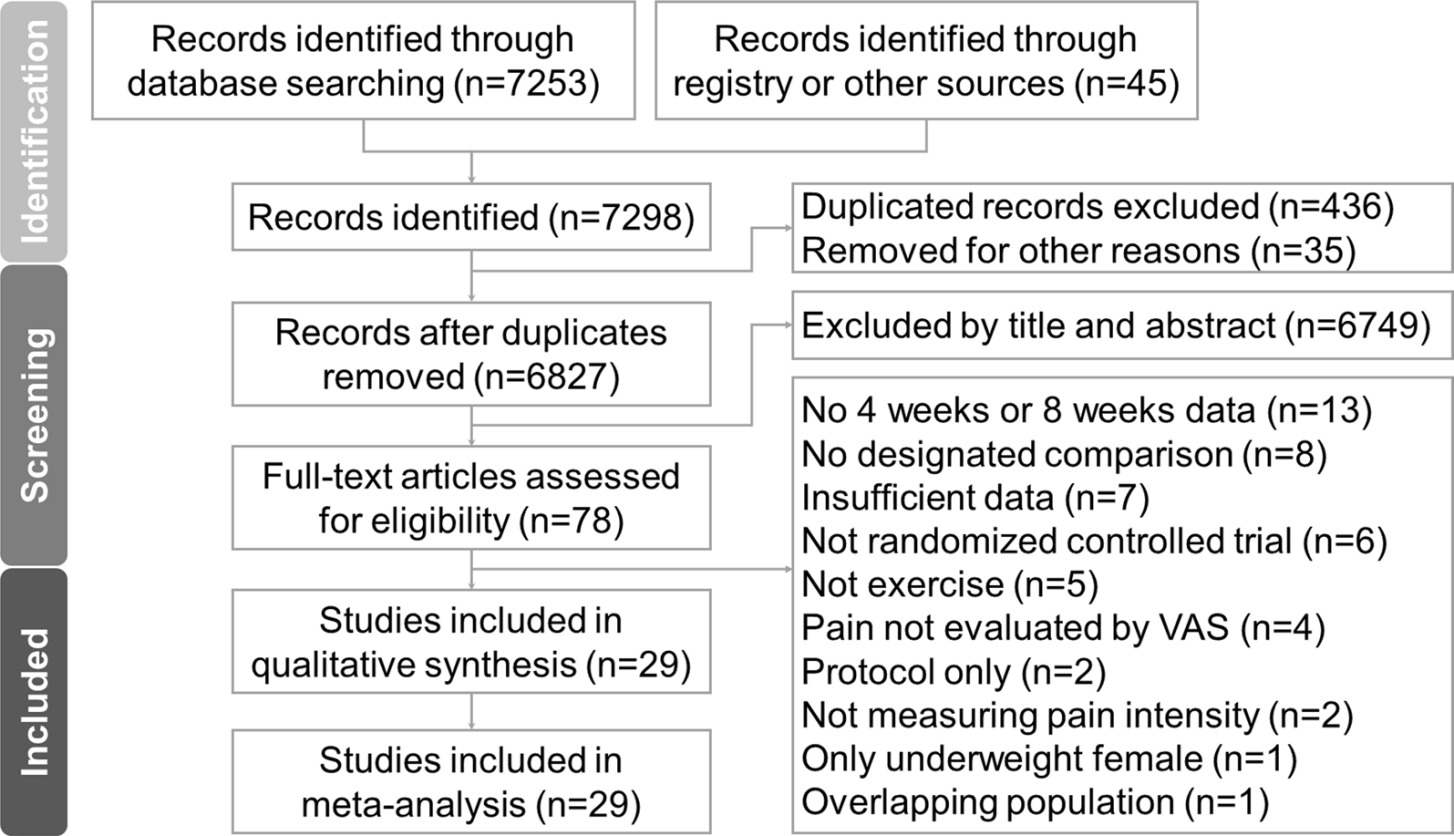
Flow diagram for the study selection process based on the Preferred Reporting Items for Systematic Reviews and Meta-analyses (PRISMA) guidelines

The PRISMA flowchart illustrating the literature search process can be observed in Fig. 1. Additionally, the PRISMA NMA extension’s checklist has been provided in Additional file 1: Table S1. The total number of articles retrieved from various databases is detailed in Additional file 1: Table S2. After the removal of duplicate articles and the exclusion of non-relevant articles through a thorough examination of titles and abstracts, a total of 29 randomized controlled trials were ultimately incorporated [6, 10–12, 15–23, 38–40, 42, 45, 46, 54–63]. Detailed information regarding the articles excluded during the final stage and the reasons for their exclusion are documented in Additional file 1: Table S3 [7–9, 64–109].

**Fig. 2:**
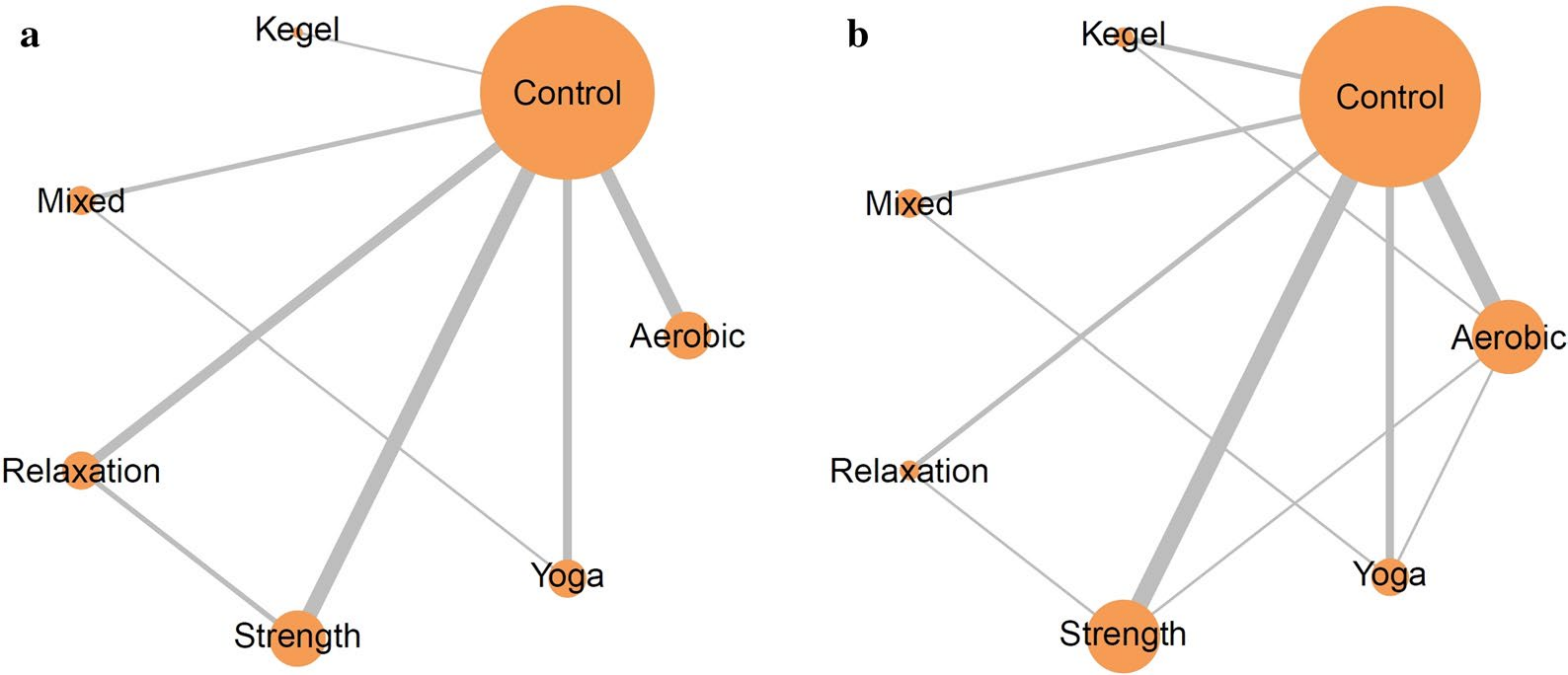
Network plots of enrolled treatments in terms of the pain intensity reduction in 4 weeks (**a**) and 8 weeks (**b**). The size of each node and thickness of each line represent the number of trials

In our analysis, the dataset encompassed a total of 1755 individuals at 4 weeks and 1808 individuals at 8 weeks, as extracted from the 29 randomized controlled trials included. The included studies categorized the exercises as follows: relaxation exercises (comprising progressive muscle relaxation and self-administered abdominal massage), strength training, aerobic activity, yoga, and Kegel maneuvers. Exercises that incorporated elements from two or more of these categories were classified as mixed exercises. The network models for the 4-week and 8-week intervals are visually depicted in Fig. 2a, b, respectively.

Among the studies encompassing women of reproductive age, 28 trials recruited participants within the age range of 15–26 years, while only one study [54] included some women beyond the age of 26. Comprehensive details concerning the countries where recruitment took place, the age range of the participants, as well as data regarding pain intensity and dropout rates, can be found in Table 1.

**Table 1:** Summary of the included trials investigating the effect of exercise to reduce pain intensity in primary dysmenorrhea

First author & Year	Study country	Included age^1^	Participants in nodes	4-week pain intensity change^2^	8-week pain intensity change^2^	8-week dropouts^3^	Notes
Abbaspour et al. 2004 [39]	Iran	15–18	97 Strength	− 3.96 ± 1.45	− 5.75 ± 1.41	0/97	The twice-daily 20-min exercise, including typical strength training exercises such as heel raise and squat, significantly reduced menstrual pain intensity at both 4 and 8 weeks as compared with the control group, especially over 8 weeks.
			45 Control	0.16 ± 0.62	0.16 ± 0.62	0/45	
Rakhshaee 2011 [10]	Iran	18–22	50 Yoga	− 3.47 ± 1.61	− 4.27 ± 1.72	10/60	Yoga for 20 min three times a week significantly reduced menstrual pain at 4 and 8 weeks as compared to the control group. The study used a VAS ranging from 0 to 3, with scores multiplied by 10/3 to convert to a 10-cm scale.
			42 Control	0.34 ± 1.35	0.10 ± 1.33	18/60	
Sakuma et al. 2012 [54]	Japan	20–64	67 Yoga	− 1.92 ± 1.73			The daily 8-min yoga practice significantly reduced menstrual pain compared to a control group over 4 weeks. The study used a 100 mm VAS, with scores divided by 10 to convert to a 10-cm scale.
			31 Control	− 0.55 ± 2.21			
Salehi et al. 2012[55]	Iran	18–25	20 Strength		− 4.88 ± 1.41	0/20	The three-times-per-week 60-min Pilates class significantly reduced menstrual pain compared to a control group over 8 weeks. Pilates is classified as a strength training exercise since it is designed to increase muscle strength [44]. The article is written in Persian with an English abstract provided, and the numbers and protocols are presented in Persian and have been translated.
			20 Control		0.05 ± 1.01	0/20	
Shahr-jerdy et al. 2012[45]	Iran	15–17	124 Strength		− 2.77 ± 1.22	0/124	The 10-min exercise twice daily significantly reduced menstrual pain compared to the control group over 8 weeks. The stretching exercise protocol in the study includes typical strength training exercises such as abdominal crunch, squat, and heel raise. The protocol requires stopping during menstruation.
			55 Control		− 0.61 ± 0.93	0/55	
Kaur et al. 2013 [22]	India	19–25	16 Kegel	− 2.60 ± 1.27	− 3.78 ± 1.38	0/16	Slow Kegel exercise with 90 contractions, each with 5–10 s of hold time. Fast Kegel exercise involves 90 quick contractions. Both exercises are performed around 3 times a week. The slow and fast Kegel exercises were combined instead of treating fast Kegel as a single arm in the node. Although Kegel exercises reduced menstrual pain compared to a control group over 4 and 8 weeks, the reduction was statistically significant only over 8 weeks, not 4 weeks.
			8 Control	− 1.53 ± 1.51	− 0.66 ± 1.03	0/8	
Reyhani et al. 2013 [56]	Iran	18–22	45 Aerobic		− 0.75 ± 1.31	0/45	Coach-assisted brisk walking for 30 min during the first 3 days of menstruation significantly reduced menstrual pain compared to the control group over 8 weeks. The article is written in Persian with an English abstract provided. The numbers and protocols are presented in Persian and have been translated.
			45 Control		0.14 ± 1.00	0/45	
Rezvani et al. 2013 [57]	Iran	18–25	20 Aerobic	− 2.38 ± 0.65	− 2.98 ± 0.93	0/20	Walking and running in water for 40–45 min three times per week significantly reduced menstrual pain compared to the control group over both 4- and 8-week periods. The target heart rate was 60–80% of the maximum. The authors used a 5-cm VAS, which has been converted to a 10-cm VAS by multiplying all scores by 2.
			20 Control	0.20 ± 0.98	− 0.08 ± 1.00	0/20	
Siahpour et al. 2013 [58]	Iran	20–25	20 Aerobic		− 3.30 ± 1.04	0/20	Both aerobic and yoga classes are 60 min per session, three times per week. Both exercises significantly reduced menstrual pain compared to the control group over 8 weeks. The article is written in Persian with an English abstract provided. The numbers and protocols are presented in Persian and have been translated.
			20 Yoga		− 2.65 ± 1.17	0/20	
			20 Control		0.05 ± 0.79	0/20	
Gamit et al. 2014 [42]	India	18–25	15 Strength	− 2.20 ± 0.79			The twice daily, 6 days per week short 9-exercise protocol significantly reduced menstrual pain compared to a control group over 4 weeks. The stretching exercise protocol in the study includes typical strength training exercises such as abdominal crunch, good morning, squat, and heel raise.
			15 Control	− 0.10 ± 0.99			
Azima et al. 2015 [16]	Iran	18–22	32 Relaxation	− 2.79 ± 1.57	− 3.61 ± 1.17	2/34	Arm 1: The participant self-administered effleurage massage for 30 min on the first 2 days of menstruation after explaining the procedure. Arm 2: Isometric exercises involving abdominal, pelvic, and femoral muscles as strength training, performed twice a day, 5 days a week. Both arms significantly reduced menstrual pain compared to the control group in the 4 and 8-week periods.
			24 Strength	− 1.94 ± 1.61	− 2.41 ± 1.27	10/34	
			28 Control	0.33 ± 1.26	0.06 ± 1.27	6/34	
Ortiz et al. 2015 [11]	Mexico	18–22	83 Mixed	− 0.10 ± 0.08	− 2.82 ± 0.08	9/92	The three-times-per-week 50-min physiotherapy intervention includes strength training, aerobic activity, and relaxation exercise, and is thus classified as a mixed category. It significantly reduced menstrual pain compared to a control group over 8 weeks, but not 4 weeks.
			77 Control	− 0.32 ± 0.09	− 0.26 ± 0.09	13/90	
Patel et al. 2015 [46]	India	17–25	60 Strength		− 4.12 ± 0.90	0/60	The twice-daily, three times a week short exercise protocol includes typical strength training exercises such as the squat and heel raise. It significantly reduced menstrual pain intensity compared to an control group over an 8-week period.
			60 Control		− 0.22 ± 0.90	0/60	
Heidarianpour et al. 2016 [59]	Iran	21–26	10 Aerobic		− 2.19 ± 0.23	0/10	The intervention involves moderate-intensity aerobic exercise on a treadmill for 30 min, with the heart rate gradually reaching 75–85% of the maximum. Training frequency was not specified. It significantly reduced menstrual pain intensity compared to a control group over an 8-week period. The article is written in Persian with an English abstract provided. The numbers and protocols are presented in Persian and have been translated.
			10 Control		− 0.10 ± 0.22	0/10	
Nasri et al. 2016 [60]	Iran	15–17	15 Aerobic		− 1.40 ± 1.06	0/15	Aerobic exercise is a 45-min three-times per week protocol, reaching 65–70% of the maximum heart rate. Kegel exercise is performed for 15 min every day. Both exercises significantly reduced menstrual pain intensity compared to a control group over a period of 8 weeks. The article is written in Persian with an English abstract provided. Numbers and protocols are presented in Persian and translated.
			15 Kegel		− 1.94 ± 0.90	0/15	
			15 Control		0.33 ± 1.10	0/15	
Saleh and Mowafy 2016 [18]	Egypt	18–23	88 Strength	− 1.95 ± 1.60	− 2.76 ± 1.18	12/100	The stretching exercise contains typical strength training exercises such as squat and heel raise, performed for 10 min, three times a day, 3 days per week. The core strengthening exercise contains typical strength training exercises such as abdominal crunch and plank, performed for 20 min, three times per day, 4 days per week. The two groups were combined into one strength training arm. The combined strength training group significantly reduced menstrual pain intensity compared to a control group over both 4- and 8-week periods.
			38 Control	0.36 ± 1.08	0.24 ± 0.94	12/50	
Shah et al. 2016 [61]	India	17–23	20 Strength		− 1.70 ± 0.73	0/20	The stretching exercise protocol in the study includes typical strength training exercises such as good morning, squat, and heel raise. The 10-min twice-per-day 4-day-per-week protocol significantly reduced menstrual pain intensity compared to an control group over an 8-week period.
			20 Control		− 0.10 ± 0.48	0/20	
Tharani et al. 2018 [62]	India	17–23	15 Strength		− 1.60 ± 0.69	0/15	Arm 1: Exercise for 45 min, 3 days per week. The stretching exercise protocol in the study includes typical strength training exercises such as good morning, squat, and heel raise. Arm 2: Aerobic exercise for 45 min, 3 days per week. Both arms showed reduced menstrual pain intensity over 8 weeks with the aerobic group significantly outperforming the strength training group.
			15 Aerobic		− 2.80 ± 0.55	0/15	
Akbas and Erdem 2019 [63]	Turkey	18–25	18 Aerobic	− 1.30 ± 1.87			Supervised 50-min three times per week aerobic fitness significantly reduced the intensity of menstrual pain compared to a control group over a 4-week period.
			19 Control	− 0.03 ± 1.68			
Chen and Hu 2019 [6]	Taiwan	20–22	105 Strength	− 1.07 ± 1.71			This posture-strengthening exercise protocol, as the authors described, is performed for 50 min, three times per week. It significantly reduced the intensity of menstrual pain compared to a control group over a 4-week period.
			106 Control	− 0.16 ± 1.72			
Heidarimoghadam et al. 2019 [40]	Iran	18–24	43 Aerobic	− 1.48 ± 1.07	− 2.53 ± 0.97	0/43	The aerobic intervention involves different endurance exercises to reach 40–60% of the maximum heart rate. The exercise duration is gradually increased from 20 to 47 min over three sessions per week. It significantly reduced menstrual pain intensity compared to a control group over 4 and 8-week periods.
			43 Control	0.31 ± 1.31	0.18 ± 1.25	0/43	
Kannan et al. 2019 [19]	New Zealand	18–43	32 Aerobic	− 0.57 ± 0.85			The supervised treadmill aerobic exercises, including a 10-min warm-up and 20-min cool-down period, are performed at 70–85% of the maximum heart rate, three times per week. It significantly reduced menstrual pain intensity compared to a control group at the 4-week time point.
			33 Control	− 0.03 ± 0.83			
Samy et al. 2019 [38]	Egypt	18–25	49 Aerobic	− 2.39 ± 0.74	− 3.39 ± 0.72	0/49	The 60-min Zumba aerobic dance was performed twice per week and significantly reduced the intensity of menstrual pain compared to a control group over a period of 4 and 8-week period.
			49 Control	0.53 ± 0.71	0.39 ± 0.72	0/49	
Kirmizigil and Demiralp 2020 [21]	Turkey	18–35	14 Mixed	− 2.40 ± 1.09	− 5.10 ± 1.01	0/14	The mixed intervention includes stretching, yoga, core strengthening, pelvic exercises, and Kegel exercises. Each session lasts 45 min, performed three times a week. It significantly reduced menstrual pain intensity compared to a control group in the 4- and 8-week periods, especially 8 weeks.
			14 Control	− 0.30 ± 0.98	− 0.20 ± 0.98	0/14	
Çelik and Apay 2021 [12]	Turkey	17–23	64 Relaxation	− 2.02 ± 1.40	− 3.72 ± 1.45	33/97	The progressive muscle relaxation is performed for 30 min, at least three times per week. It reduced menstrual pain intensity compared to a control group over both 4- and 8-week periods significantly.
			60 Control	− 0.04 ± 1.53	− 0.05 ± 1.52	37/97	
Kirca and Çelik 2021 [20]	Turkey	18–24	30 Yoga	− 0.50 ± 0.33	− 1.70 ± 0.32	2/32	Yoga in a 60-min once a week group significantly reduced menstrual pain intensity compared to a control group during the 4- and 8-week periods, especially 8 weeks.
			30 Control	− 0.04 ± 0.42	− 0.07 ± 0.43	5/35	
Ozturk et al. 2022 [17]	Turkey	18–20	22 Strength	− 1.10 ± 1.01			Arm 1: Short exercise, performed three times per day during the first 3 days of the menstrual cycle. Arm 2: The participant herself performed an effleurage massage, lasting 10 min, during the first 3 days of the menstrual cycle. Both arms showed non-significant reduction in the intensity of menstrual pain in 4 week.
			22 Relaxation	− 1.40 ± 1.03			
			19 Control	− 0.60 ± 1.11			
Yildiz and Acaroğlu 2022[15]	Turkey	18–22	50 Relaxation	− 7.88 ± 1.57			The intervention involves progressive muscle relaxation and self-massage, with progressive relaxation performed for 30 min and massage for 15 min. The intervention is performed three times per day during the first several days of menstrual pain. It significantly reduced the intensity of menstrual pain compared to a control group over a 4-week period.
			47 Control	0.34 ± 1.91			
Yosri et al. 2022 [23]	Egypt	19–25	30 Yoga	− 2.07 ± 0.79	− 3.91 ± 0.73	0/30	Arm 1: Yoga only. Arm 2: Yoga plus three different types of squats, combined into one mixed exercise arm. The squats are performed for 1 × 10 to 5 × 10 repetitions, with a 30–60 s rest period between sets. The intervention is performed three times a week. The plus-squat group significantly reduced menstrual pain intensity compared to yoga-only group over both 4- and 8-week periods.
			90 Mixed	− 2.43 ± 0.79	− 4.82 ± 0.71	0/90	

^1^The age range included in the Methods section is displayed first. If this information is not provided, the range is estimated from the description of the inclusion criteria (e.g., 18–22 for university students) or is rounded from the mean ± 2 standard deviations of the analyzed participants mentioned in the Results section

^2^Pain intensity was measured using a 10-cm visual analogue scale. For studies using a 3- or 5-cm scale, the numbers were linearly converted to a 10-cm scale. Specifically, the means and standard deviations were multiplied by 10/3 or 10/5, respectively

^3^The data are presented as dropout numbers/intention-to-treat numbers

### Methodological Quality of the Included Studies

With respect to the overall methodological quality of the included studies, our evaluation revealed that 37.9% of them exhibited a low risk of bias, while 58.6% were associated with some risk of bias. A minor proportion, accounting for 3.4%, exhibited a high risk of bias, as depicted in Additional file 1: Figure S1. Specifically, we identified eighteen studies [10, 12, 17, 22, 39, 40, 42, 45, 46, 55–63] that demonstrated some risk of bias in the randomization process due to insufficient details regarding randomization procedures and allocation concealment, or the use of an allocation sequence that could be readily predicted [12, 17]. Moreover, one study [46] received a high-risk rating concerning selective reporting, as it gathered data on pain duration without subsequently reporting them. Detailed information concerning the assessment of the risk of bias is comprehensively documented in Additional file 1: Table S4.

### Primary Outcome: Pain Intensity

**Fig. 3:**
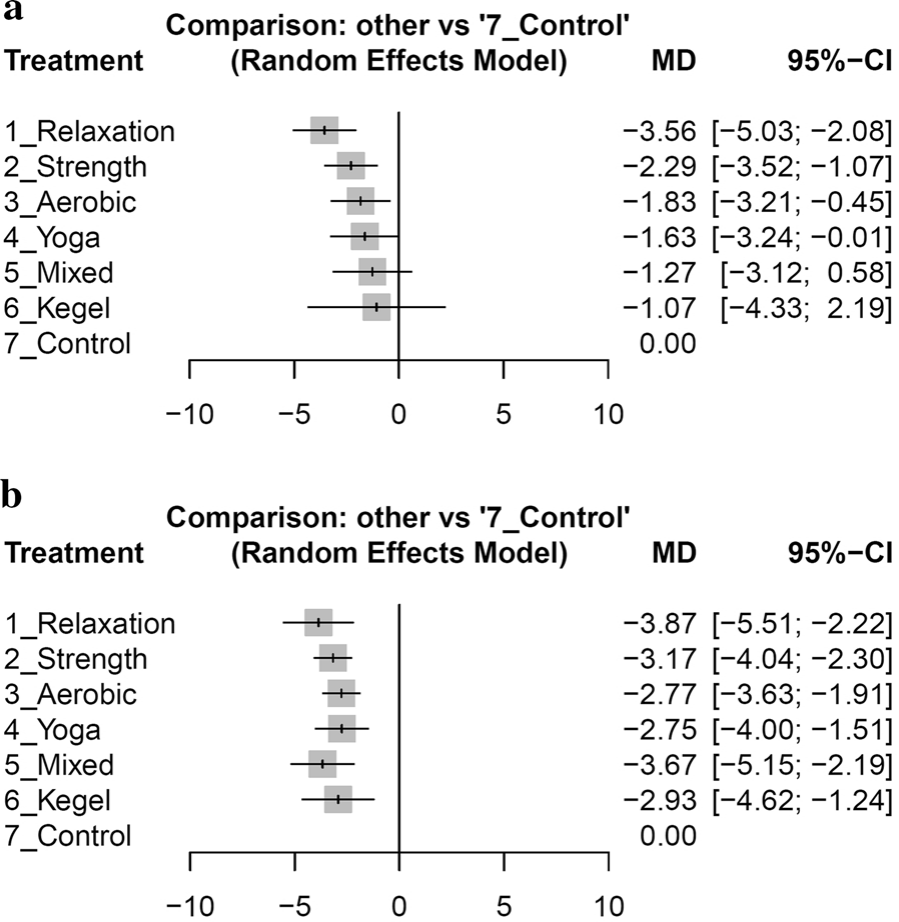
Forest plots of the mean difference (MD) between various types of exercise and control group for pain reduction in primary dysmenorrhea patients at 4 weeks (**a**) and 8 weeks (**b**)

Following a 4-week intervention period, all exercise modalities demonstrated varying degrees of pain intensity reduction in comparison to the control group, with three of them showing statistically significant reductions, and one exhibiting an effect of borderline significance. Based on the ranking of point estimates, the three exercises that exhibited statistically significant reductions in pain intensity were relaxation exercises (− 3.56; 95% CI − 5.03 to − 2.08), strength training (− 2.29; 95% CI − 3.52 to − 1.07), and aerobic activity (− 1.83; 95% CI − 3.21 to − 0.45), whereas yoga demonstrated an effect of borderline significance (− 1.63; 95% CI − 3.24 to − 0.01), as illustrated in Fig. 3a.

Upon completion of the 8-week intervention period, it is noteworthy that all exercise modalities exhibited effect sizes with 95% CIs that did not intersect with 0, indicating statistically significant reductions in menstrual pain. Based on the point estimates of effect sizes, the interventions were ranked in the following order (as depicted in Fig. 3b): relaxation exercise (− 3.87; 95% CI − 5.51 to − 2.22), mixed exercise (− 3.67; 95% CI − 5.15 to − 2.19), strength training (− 3.17; 95% CI − 4.04 to − 2.30), Kegel maneuvers (− 2.93; 95% CI − 4.62 to − 1.24), aerobic activity (− 2.77; 95% CI − 3.63 to − 1.91), and yoga (− 2.75; 95% CI − 4.00 to − 1.51). These findings suggest that all of the aforementioned exercise interventions were efficacious in reducing menstrual pain by the eighth week.

Table 2 provides a comprehensive overview of the ranking and pairwise comparisons among all intervention groups at both the 4-week and 8-week assessment points, respectively. Moreover, Additional file 1: Figure S2a, b present visual representations of the mean differences observed in the pairwise comparisons for the 4-week and 8-week analyses, respectively.

**Table 2:** Pairwise comparison and ranking of various exercise interventions for reducing menstrual pain intensity at 4 and 8 weeks

At four weeks						
**Relaxation**	− 0.57 [− 2.77, 1.63]					− 3.52 [− 5.06, − 1.97]
− 1.26 [− 2.94, 0.42]	**Strength**					− 2.04 [− 3.30, − 0.79]
− 1.72 [− 3.74, 0.30]	− 0.46 [− 2.31, 1.39]	**Aerobic**				− 1.83 [− 3.21, − 0.45]
− 1.93 [− 4.11, 0.25]	− 0.67 [− 2.69, 1.36]	− 0.21 [− 2.33, 1.92]	**Yoga**	0.36 [− 2.68, 3.40]		− 1.87 [− 3.66, − 0.09]
− 2.29 [− 4.65, 0.08]	− 1.02 [− 3.24, 1.19]	− 0.56 [− 2.87, 1.74]	− 0.36 [− 2.42, 1.71]	**Mixed**		− 0.90 [− 3.07, 1.27]
− 2.49 [− 6.06, 1.09]	− 1.22 [− 4.70, 2.26]	− 0.76 [− 4.30, 2.77]	− 0.56 [− 4.19, 3.08]	− 0.20 [− 3.94, 3.54]	**Kegel**	− 1.07 [− 4.33, 2.19]
− 3.56 [− 5.03, − 2.08]	− 2.29 [− 3.52, − 1.07]	− 1.83 [− 3.21, − 0.45]	− 1.63 [− 3.24, − 0.01]	− 1.27 [− 3.12, 0.58]	− 1.07 [− 4.33, 2.19]	**Control**

At eight weeks						
**Relaxation**		− 1.20 [− 3.70, 1.30]				− 3.67 [− 5.43, − 1.91]
− 0.20 [− 2.41, 2.02]	**Mixed**			− 0.91 [− 3.35, 1.53]		− 3.68 [− 5.43, − 1.93]
− 0.69 [− 2.44, 1.05]	− 0.50 [− 2.21, 1.22]	**Strength**			1.20 [− 1.26, 3.66]	− 3.42 [− 4.35, − 2.49]
− 0.94 [− 3.30, 1.42]	− 0.75 [− 2.99, 1.50]	− 0.25 [− 2.13, 1.64]	**Kegel**		− 0.54 [− 3.06, 1.98]	− 2.68 [− 4.49, − 0.87]
− 1.12 [− 3.18, 0.94]	− 0.92 [− 2.56, 0.72]	− 0.42 [− 1.93, 1.09]	− 0.18 [− 2.26, 1.91]	**Yoga**	0.65 [− 1.86, 3.16]	− 2.88 [− 4.30, − 1.45]
− 1.10 [− 2.94, 0.75]	− 0.90 [− 2.59, 0.79]	− 0.40 [− 1.55, 0.75]	− 0.15 [− 1.93, 1.62]	0.02 [− 1.41, 1.46]	**Aerobic**	− 2.50 [− 3.43, − 1.56]
− 3.87 [− 5.51, − 2.22]	− 3.67 [− 5.15, − 2.19]	− 3.17 [− 4.04, − 2.30]	− 2.93 [− 4.62, − 1.24]	− 2.75 [− 4.00, − 1.51]	− 2.77 [− 3.63, − 1.91]	**Control**

Values are mean differences with 95% confidence intervals. The estimates from pairwise meta-analyses are located above the diagonal line, while the estimates from network meta-analyses are located below the diagonal line

### Secondary Outcome: Difference in Risk of Dropout

**Fig. 4:**
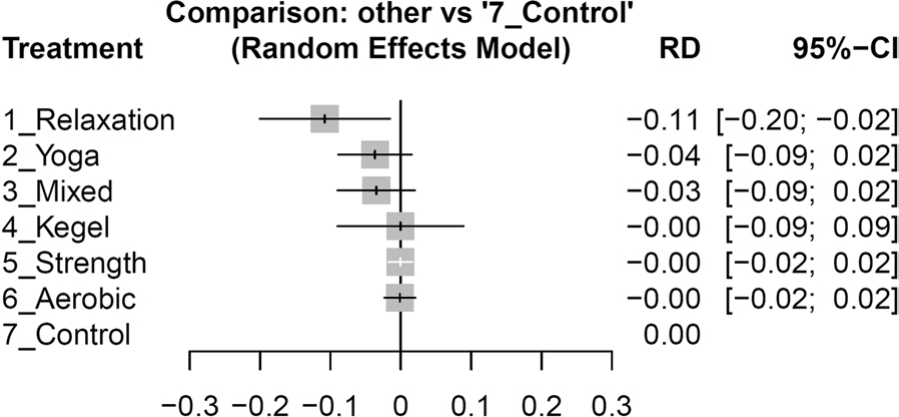
Forest plot of the risk difference (RD) of dropout rate at 8 weeks for each type of exercise compared to the control group

Through our analysis of the impact of dropouts at 8 weeks, employing the risk difference metric, we observed that five out of the six types of exercises exhibited 95% CIs that overlapped with 0. This indicates that there was no statistically significant difference in dropout rates when compared to the control group, as illustrated in Fig. 4. The sole exception was relaxation exercise, which displayed a risk difference effect size of − 0.11 (95% CI − 0.20 to − 0.02), signifying an 11% lower risk of dropout in comparison to the control group. These findings suggest that among all the exercises and the control group, relaxation exercise had the lowest risk of dropout.

Additional file 1: Figure S3 presents the differences in risk of dropout between various types of exercises as well as between the exercise groups and the control group for the studies with available data at 8 weeks.

### Inconsistency Test

Utilizing the network generated by the interconnected nodes, we conducted both direct and indirect comparisons and subjected them to statistical assessment to ascertain consistency. The outcomes of the pertinent tests for pain intensity at 4 weeks and 8 weeks can be reviewed in Additional file 1: Table S5, while the results for dropout rates at 8 weeks are detailed in Additional file 1: Table S6. Importantly, all available comparisons yielded *p* values exceeding 0.05, signifying a lack of evidence supporting inconsistency between direct and indirect comparisons.

### Sensitivity Analysis

A sensitivity analysis was undertaken by adjusting the pre-post correlation coefficient from 0.8 to 0.5, followed by a recalculation of the network comparisons at both four and 8 weeks, as illustrated in Additional file 1: Figure S4a, b. Encouragingly, our findings revealed that the direction of effect sizes, ranking, and interpretation of the results remained consistent with those obtained using a coefficient of 0.8, as observed in Fig. 3a, b. Additionally, it is worth noting that the actual numerical differences between the two coefficients were all less than 0.05, a value that is considered clinically insignificant on a 10 cm VAS.

### Publication Bias

Additional file 1: Figure S5 shows the funnel plot for publication bias. The Egger’s test yielded a *p* value of 0.20, indicating no significant publication bias.

## Discussion

### Main Findings and Clinical Implications

Based on the findings of our study, it is evident that all types of exercises were efficacious in reducing menstrual pain when compared to the control group at the 8-week mark. Furthermore, there were no discernible differences in dropout risk between any of the exercise modalities, except for relaxation exercise, when compared to the control group. Relaxation exercises emerged as the most effective approach for pain reduction, as evidenced by a 10-cm VAS score of − 3.56 (95% CI − 5.03 to − 2.08) at 4 weeks and − 3.87 (95% CI − 5.51 to − 2.22) at 8 weeks. Additionally, relaxation exercise exhibited a notably lower dropout rate of − 0.11 (95% CI − 0.20 to − 0.02) in comparison to other exercise modalities and the control group, further underscoring its effectiveness.

This research has significant value for women who are considering exercise as a means to alleviate menstrual pain. It offers valuable insights into the effectiveness of various exercise modalities in reducing menstrual pain and provides information about the expected timeline for experiencing significant improvements. For instance, women interested in strength training can anticipate experiencing relief as early as 4 weeks into the regimen. Conversely, those opting for Kegel exercises should be prepared for a longer timeframe, with noticeable effects potentially taking up to two months to manifest. In cases where there is no specific preference, selecting relaxation exercises is likely to be associated with a lower likelihood of discontinuation and a higher probability of achieving early results.

### Significance of the Findings Compared to Existing Literature

The studies by Matthewman et al. [13] and Carroquino-Garcia et al. [14] conducted pairwise meta-analyses to explore the impact of physical activity on primary dysmenorrhea. They both found that exercise could lead to a reduction in pain intensity, with Matthewman et al. reporting a decrease of − 1.89 (95% CI − 2.96 to − 1.09) on a 10 cm VAS scale and Carroquino-Garcia et al. indicating a reduction of − 1.86 (95% CI − 3.17 to − 0.55). However, these studies faced limitations due to grouping all types of exercise together, resulting in high heterogeneity among the included trials.

In contrast, our network meta-analysis addresses this limitation by separately evaluating different types of exercises. This approach allows us to provide a more nuanced understanding of the preferable exercise type and ideal duration for alleviating menstrual pain, which previous traditional meta-analyses [32–34, 36] were unable to achieve due to their reliance on traditional meta-analysis methods.

### Possible Physiological and Psychological Mechanisms Underlying the Observed Results

Menstrual pain is primarily attributed to the secretion of prostaglandins, which induces uterine contractions, diminishes uterine blood flow, and heightens pain sensitivity [1]. Exercise exerts multiple physiological effects, including enhanced blood circulation to various body regions [110], dilation of blood vessels [111], and an elevation of pain threshold [112, 113]. Additionally, exercise promotes the release of endorphins [4, 5], modulates macrophage polarization, shifting from the production of inflammatory cytokines like IL-1β to anti-inflammatory cytokines such as IL-10 [114], thereby yielding an analgesic effect that contributes to pain alleviation. The psychological aspects of exercise play a crucial role in pain sensation reduction, encompassing mechanisms such as distraction, cognitive focus redirection [115], stress mitigation, mood enhancement [116], and the cultivation of a sense of control and empowerment [117]. These combined physiological and psychological effects may elucidate the varying efficacy of different exercise modalities, particularly when employed in more extended interventions, such as those spanning approximately 8 weeks.

Relaxation exercise emerged as the most efficacious physical activity for pain reduction, displaying significant effectiveness at both the 4 and 8-week points. This intervention encompasses techniques such as progressive muscle relaxation [118] and abdominal massage [119], which are commonly utilized nursing strategies for managing acute post-operative pain [119]. The relaxation effect elicited by these techniques contributes to a reduction in pain sensitivity [120].

Strength training and aerobic activity, ranked as the second and third most effective exercises, respectively, represent moderate to high-intensity exercise modalities that exert a more pronounced influence on pain sensitivity and overall physical well-being. In contrast, mixed exercises may exhibit reduced effectiveness, potentially attributable to the heterogeneity of interventions or challenges associated with adhering to relatively complex protocols. The observed non-significant effect of Kegel exercises at 4 weeks may be attributed to their low-intensity nature.

An intriguing finding in our study was the similarity in dropout rates between the exercise groups and the control group, which could be attributed to two key factors. Firstly, the exercise interventions employed in our study were characterized by their ease of practice [45], minimal time requirements [10], and high tolerability [6], making them well-suited for participants. Secondly, some individuals with primary dysmenorrhea who were allocated to the regular care (no intervention) group may have sought additional measures to alleviate their menstrual pain, leading to protocol violations and subsequent dropout. Among the included studies, certain participants opted to initiate contraceptive pill usage as a means of managing menstrual pain [20], while others explored complementary therapies [12].

### Limitations

Our study has certain limitations. The enrolled studies predominantly focused on individuals with regular menstrual cycles, a practical choice to facilitate the organization of exercise programs and follow-up assessments. Regrettably, this approach resulted in the exclusion of women with irregular menstrual cycles, a condition affecting 5.0–35.6% of reproductive-age women [121]. Nevertheless, it should be acknowledged that the inclusion of patients with irregular menstrual cycles in a clinical trial would pose significant technical challenges.

Secondly, our review encompassed 29 studies, with 28 of them enrolling participants within the age range of 15–26 years, while only one study incorporated participants over the age of 26 [54]. This composition may potentially restrict the generalizability of our findings to encompass all women. However, it is essential to note that menstrual pain typically reaches its peak severity in the initial years following menarche and gradually diminishes with advancing age [1]. Consequently, our study outcomes retain relevance and utility within the context of clinical practice.

## Conclusion

In summary, our analysis revealed that all forms of exercise yielded noteworthy reductions in pain intensity at the 8-week mark. Notably, relaxation exercise emerged as the most efficacious intervention for pain reduction at both the 4 and 8-week time points, additionally exhibiting a significantly lower dropout risk when compared to the control group.

### Supplementary Information

Additional File 1.

## Data Availability

Data are contained within the article and Supplementary Files.

## Code Availability

MetaInsight is a service provided by the Complex Reviews Support Unit that utilizes the statistical software R, with a website interface powered by R-shiny. For detailed information regarding the software codes, please contact the corresponding party. Comprehensive Meta-Analysis version 4 is a software package developed by Biostat that is available for commercial use.
