# Supplementary material for: Comparative Effectiveness of Different Exercises for Reducing Pain Intensity in Primary Dysmenorrhea: A Systematic Review and Network Meta-analysis of Randomized Controlled Trials
Source: Sports Med Open. 2024 May 30;10:63. doi: 10.1186/s40798-024-00718-4 (PMC11139836; doi:10.1186/s40798-024-00718-4)
Supplement: Supplementary file 1 — Additional File 1. [file 40798_2024_718_MOESM1_ESM.docx]

**Supplementary Information (Supplementary Figures and Tables)**

**Figure S1**


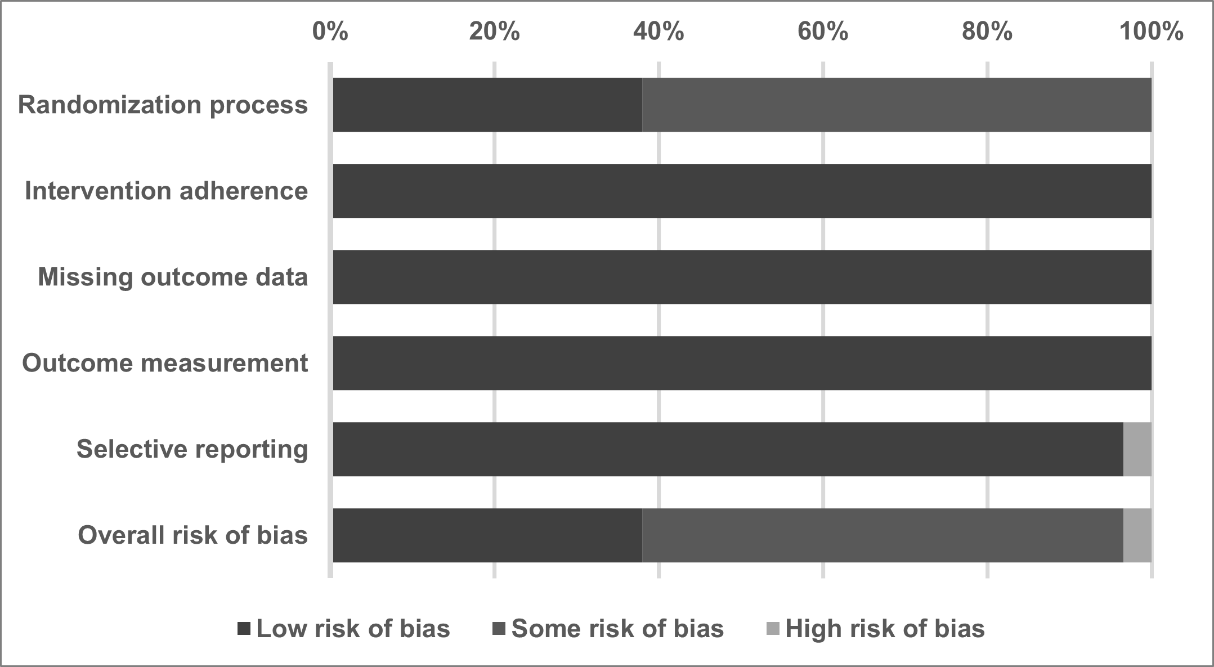


**Fig. S1** - Summary of quality assessment for the studies included in the current network meta-analysis using version 2 of the Cochrane risk-of-bias tool for randomized controlled trials. More than half of the studies received some risks in the randomization process domain. This is because most studies did not clearly describe the randomization process, or the authors used a fixed sequence that was easy to guess.

**Figure S2**

a
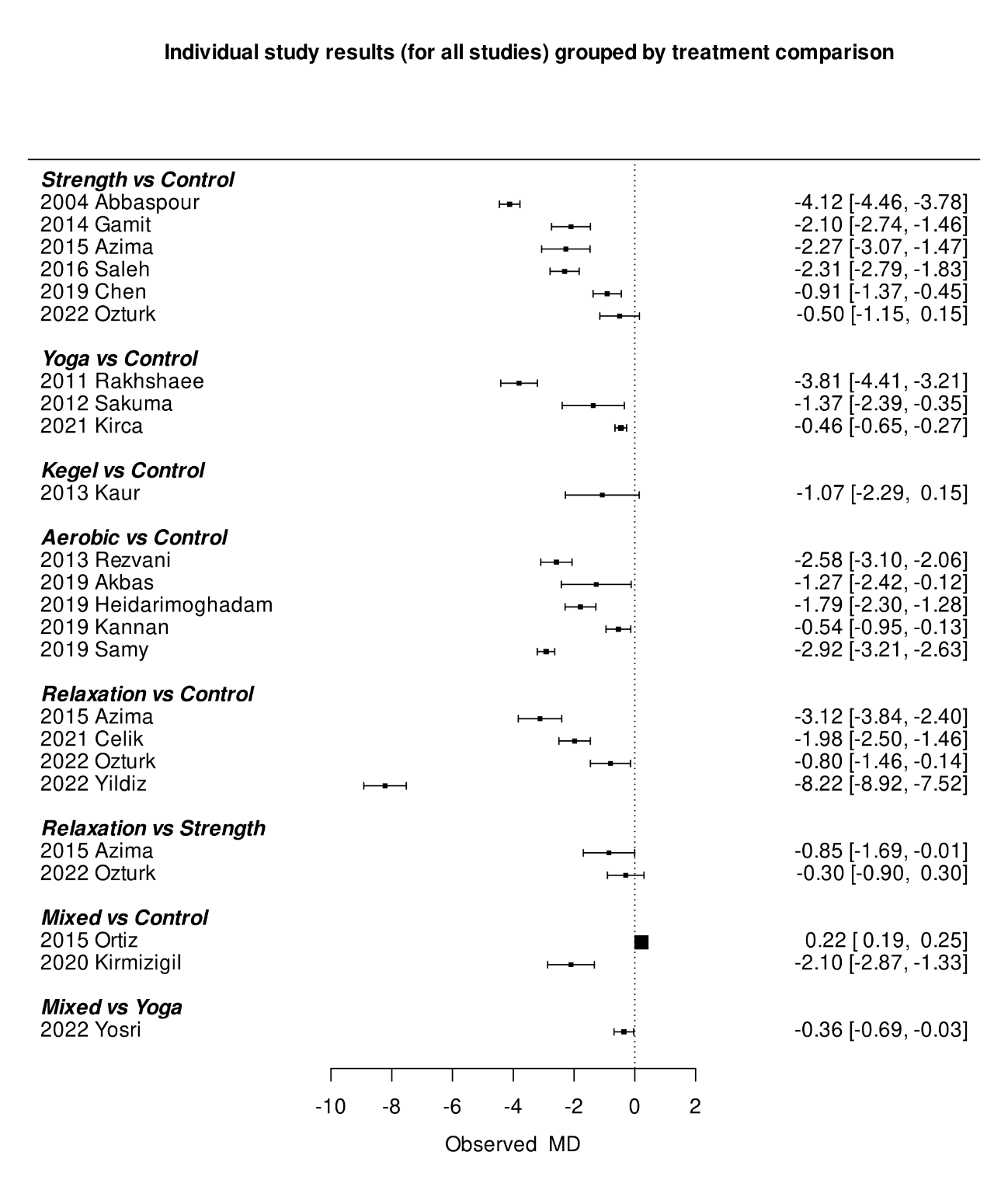


b
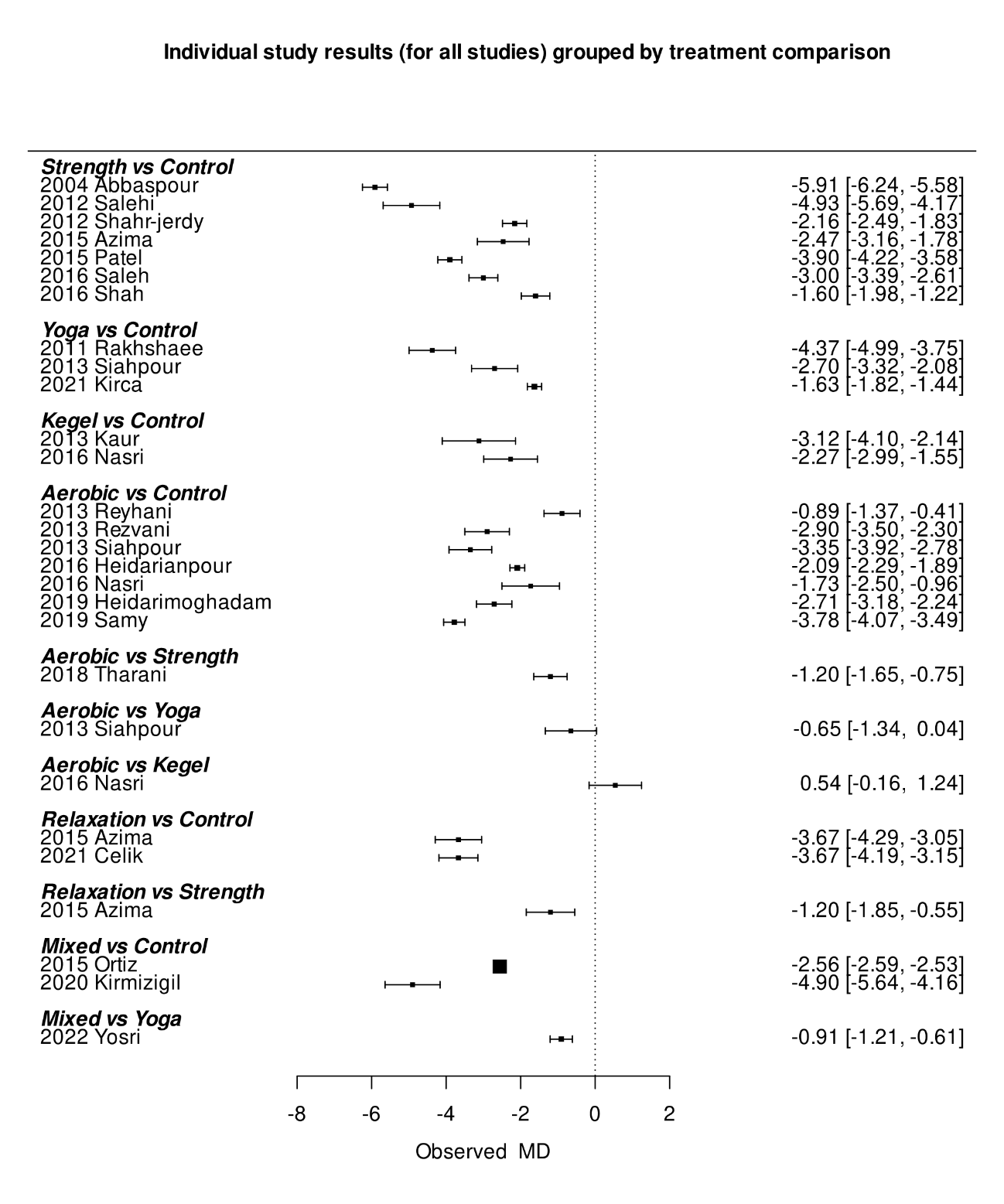


**Fig. S2** - Pair-wise comparison of the mean difference in visual analogue scale among different types of exercise in all included studies, at four weeks (a) and eight weeks (b). It can be observed that the effect sizes generally shifted to the left from four weeks to eight weeks, indicating effective pain reduction. MD: mean difference.

**Figure S3**


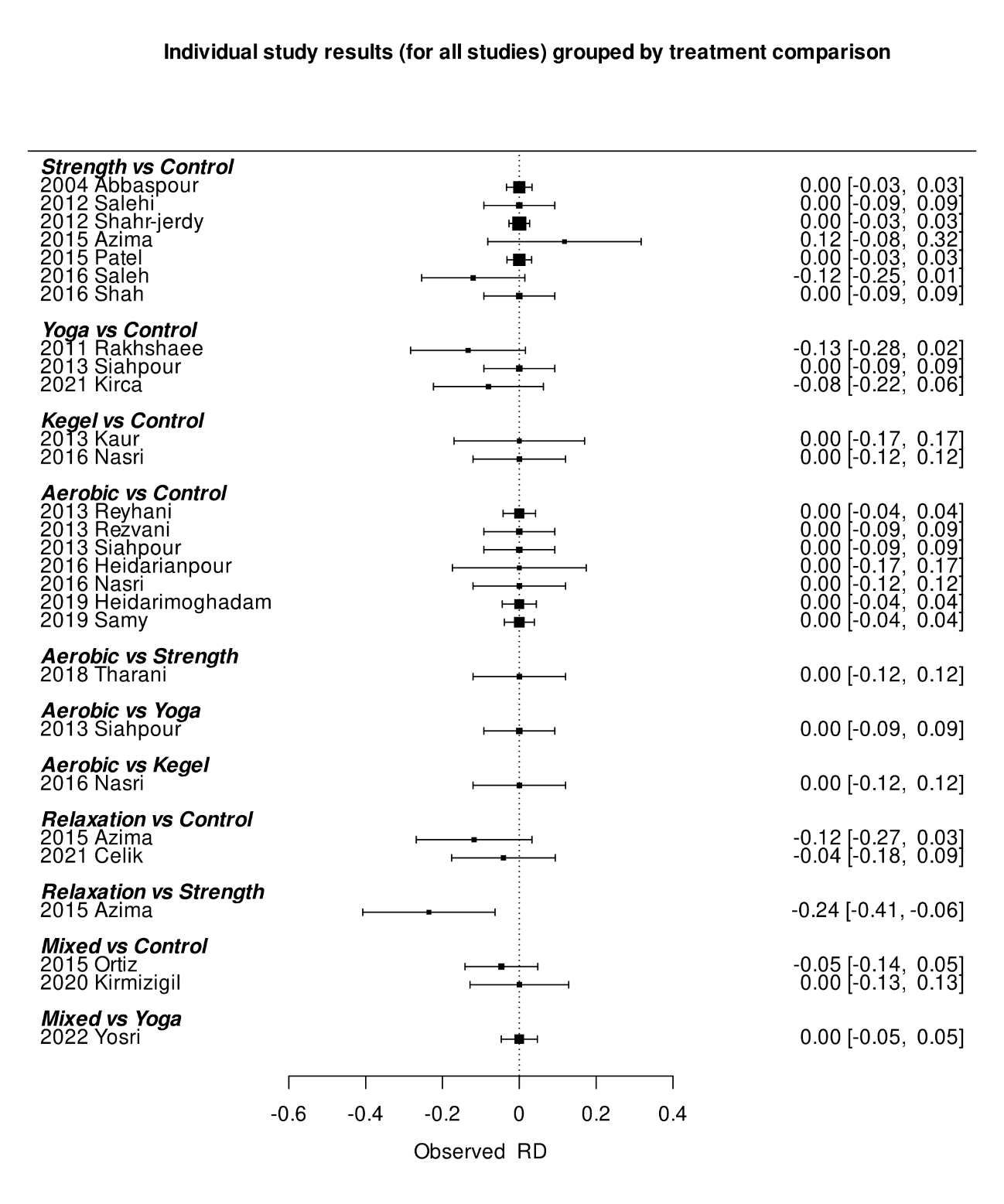


**Fig. S3** - Forest plot for the difference in risk of dropout rates at eight weeks in individual studies shows that the majority of the studies and comparisons did not reach statistical significance. Only relaxation exercise showed a significantly lower risk of dropout compared to strength training. RD: risk difference.

**Figure S4**

a
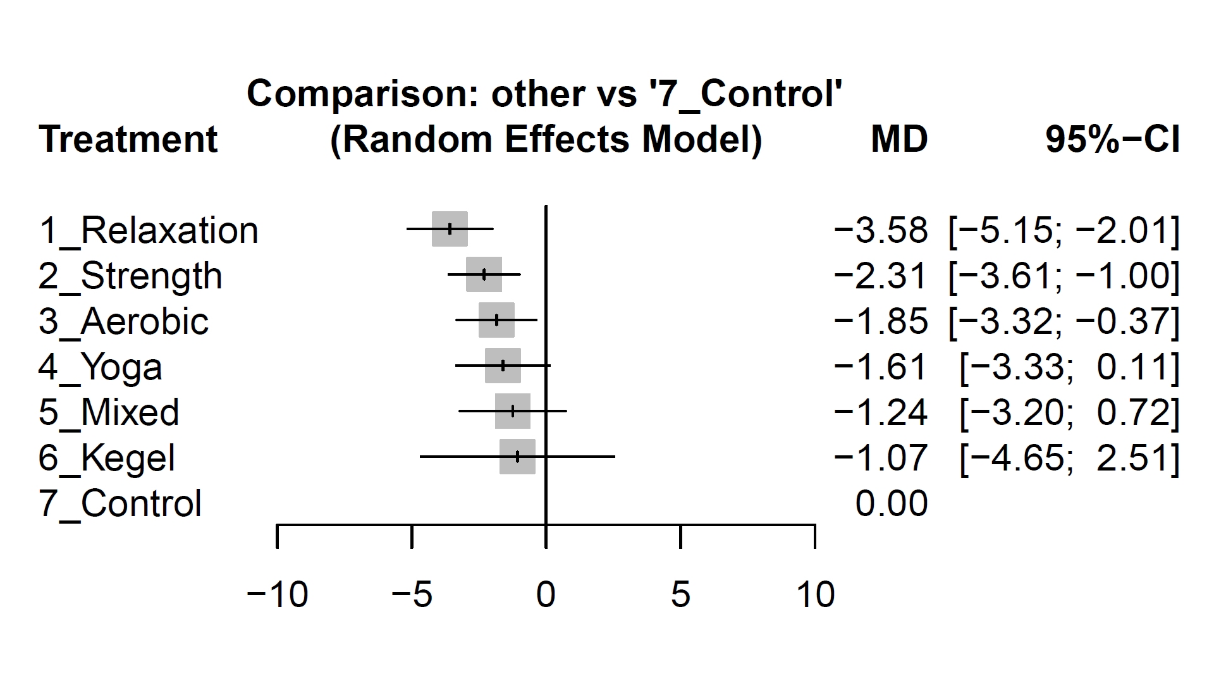


b
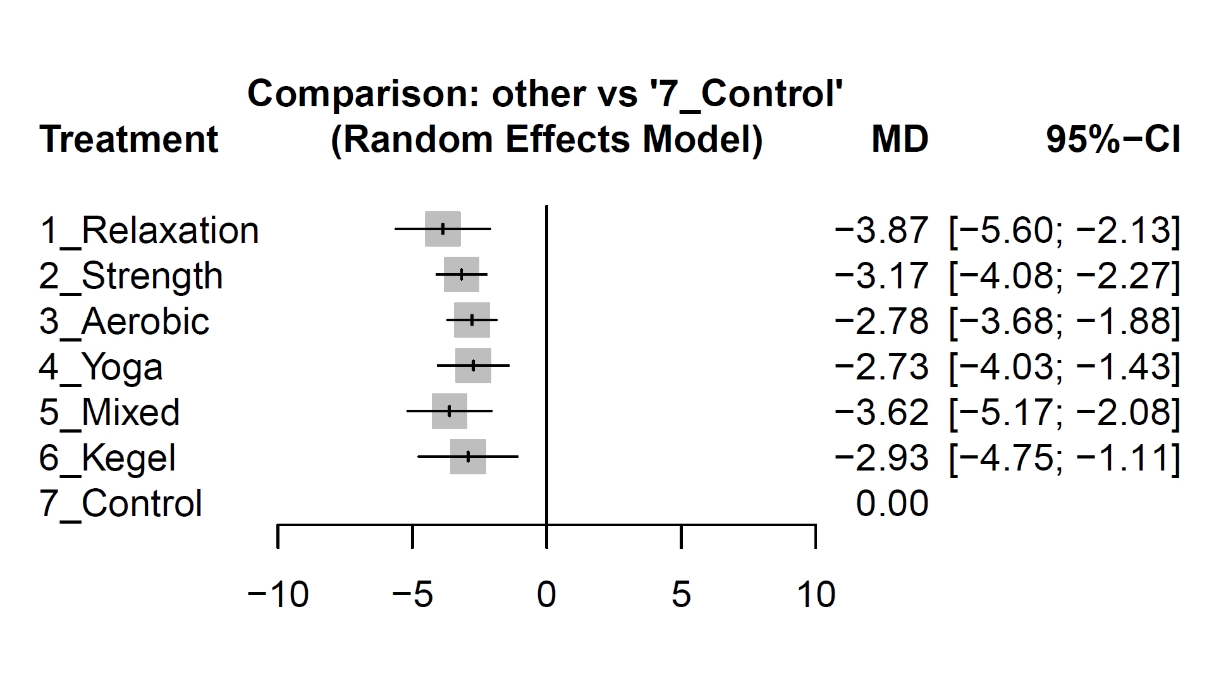


**Fig. S4** - Forest plots of different types of exercise compared to the control group in reducing pain intensity in primary dysmenorrhea patients at four weeks (a) and eight weeks (b), with pre-post correlation coefficient assumptions changed to 0.5. Compared to Figures 3a and 3b, where the pre-post correlation coefficient was assumed to be 0.8, the direction and clinical significance of the forest plots remain the same, and even the effect sizes differ only by the second decimal place. The greatest difference appears in mixed exercise at eight weeks, where the effect size compared to the control group only differs by 0.05, which is clinically negligible. Retesting with different assumptions demonstrates that the conclusions of this study remain unchanged regardless of the setting of the pre-post correlation coefficient. This indicates that the current study has passed the sensitivity analysis.

**Figure S5**


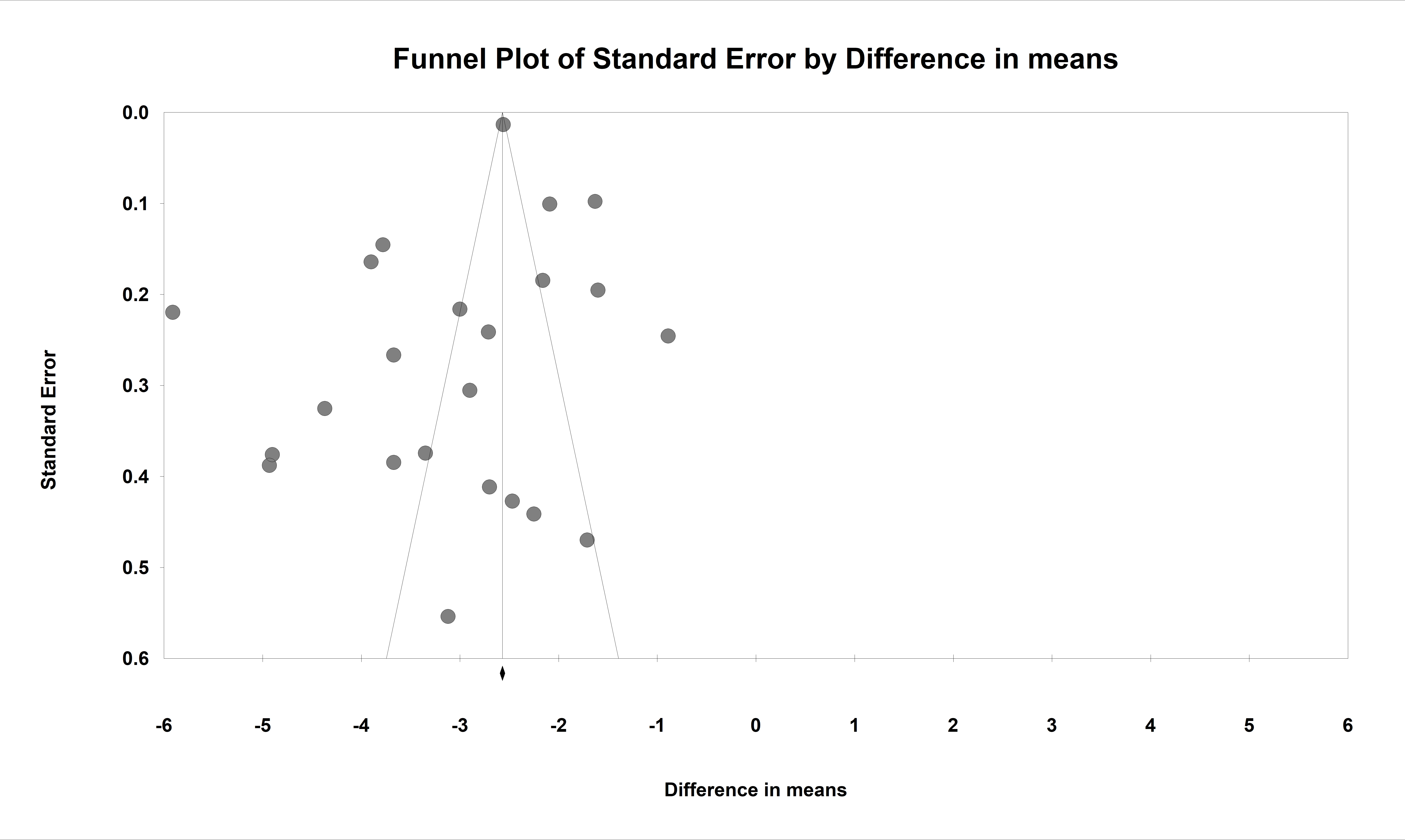


**Fig. S5** - Funnel plot of all paired comparisons involving the common comparator, control group. The Egger's test yielded a *p* value of 0.20, indicating no significant publication bias.

**Table S1** - PRISMA for network meta-analysis checklist

| **Section and Topic** | **#** | **Checklist item** | **Location** |
| --- | --- | --- | --- |
| **Title** |  |  |  |
| Title | 1 | Identify the report as a systematic review incorporating a network meta-analysis (or related form of meta-analysis). | Title |
| **Abstract** |  |  |  |
| Structured summary | 2 | Provide a structured summary including, as applicable: **Objective**: main objectives / **Methods**: data sources; study eligibility criteria, participants, and interventions; study appraisal; and synthesis methods, such as network meta-analysis. / **Results**: number of studies and participants identified; summary estimates with corresponding confidence/credible intervals; treatment rankings may also be discussed. Authors may choose to summarize pairwise comparisons against a chosen treatment included in their analyses for brevity. / **Conclusions**: limitations; conclusions and implications of findings. / **Other**: primary source of funding; systematic review registration number with registry name. | Abstract |
| **Introduction** |  |  |  |
| Rationale | 3 | Describe the rationale for the review in the context of what is already known, including mention of why a network meta-analysis has been conducted. | Introduction  1^st^, 2^nd^, and 3^rd^ paragraphs |
| Objectives | 4 | Provide an explicit statement of questions being addressed, with reference to participants, interventions, comparisons, outcomes, and study design (PICOS). | Introduction  4^th^ and 5^th^ paragraphs |
| **Methods** |  |  |  |
| Protocol & registration | 5 | Indicate whether a review protocol exists and where it can be accessed; and, if available, provide registration information, including registration number. | Materials and Methods  1^st^ paragraph |
| Eligibility criteria | 6 | Specify study characteristics (e.g., PICOS, length of follow-up) and report characteristics (e.g., years considered, language, publication status) used as criteria for eligibility, giving rationale. Clearly describe eligible treatments included in the treatment network, and note whether any have been clustered or merged into the same node (with justification). | Materials and Methods /  2.2 Inclusion and exclusion |
| Information sources | 7 | Describe all information sources (e.g., databases with dates of coverage, contact with study authors) in the search and date last searched. | Table S2 |
| Search | 8 | Present full electronic search strategy for at least one database, including any limits used, such that it could be repeated. | Table S2 |
| Study selection | 9 | State the process for selecting studies (i.e., screening, eligibility, included in systematic review, and, if applicable, included in the meta-analysis). | Materials and Methods /  2.1 & 2.2 |
| Data collection | 10 | Describe method of data extraction from reports (e.g., piloted forms, independently, in duplicate) and any processes for obtaining and confirming data. | Materials and Methods /  2.7 Data extraction |
| Data items | 11 | List and define all variables for which data were sought (e.g., PICOS, funding sources) and any assumptions and simplifications made. | Materials and Methods /  2.1 to 2.7 |
| Network geometry | S1 | Describe methods used to explore the geometry of the treatment network under study and potential biases related to it. This should include how the evidence base has been graphically summarized for presentation, and what characteristics were compiled and used to describe the evidence base to readers. | Materials and Methods /  2.3 Modeling |
| Risk of bias within | 12 | Describe methods used for assessing risk of bias of individual studies (including specification of whether this was done at the study or outcome level), and how this information is to be used in any data synthesis. | Materials and Methods /  2.4 Quality appraisal |
| Summary measures | 13 | State the principal summary measures (e.g., risk ratio, difference in means). Also describe the use of additional summary measures assessed, such as treatment rankings, as well as modified approaches used to present summary findings from meta-analyses. | Materials and Methods /  2.5 2.6 Outcomes |
| Planned methods of analysis | 14 | Describe the methods of handling data and combining results of studies for each network meta-analysis. This should include, but not be limited to: Handling of multi-arm trials; Selection of variance structure; Selection of prior distributions in Bayesian analyses; and Assessment of model fit. | Materials and Methods /  2.8 Statistical analysis |
| Assessment of inconsistency | S2 | Describe the statistical methods used to evaluate the agreement of direct and indirect evidence in the treatment network(s) studied. Describe efforts taken to address its presence when found. | Materials and Methods /  2.8 Statistical analysis |
| Risk of bias across | 15 | Specify any assessment of risk of bias that may affect the cumulative evidence. | Materials and Methods /  2.10 Publication bias |
| Additional analyses | 16 | Describe methods of additional analyses if done, indicating which were pre-specified. This may include, but not be limited to, the following: Sensitivity or subgroup analyses; Meta-regression analyses; Alternative formulations of the treatment network; and Use of alternative prior distributions for Bayesian analyses (if applicable). | Materials and Methods /  2.9 Sensitivity analysis |
| **Results** |  |  |  |
| Study selection | 17 | Give numbers of studies screened, assessed for eligibility, and included in the review, with reasons for exclusions at each stage, ideally with a flow diagram. | Results / 3.1 Study identification  Figure 1, Table S2, Table S3 |
| Network structure | S3 | Provide a network graph of the included studies to enable visualization of the geometry of the treatment network. | Figure 2a, Figure 2b |
| Network geometry | S4 | Provide a brief overview of characteristics of the treatment network. This may include commentary on the abundance of trials and randomized patients for the different interventions and pairwise comparisons in the network, gaps of evidence in the treatment network, and potential biases reflected by the network structure. | Figure 2a, Figure 2b,  Figure 2 legends |
| Study characteristics | 18 | For each study, present characteristics for which data were extracted (e.g., study size, PICOS, follow-up period) and provide the citations. | Table 1 |
| Risk of bias within | 19 | Present data on risk of bias of each study and, if available, any outcome level assessment. | Table S4, Figure S1,  3.2 Methodological quality |
| Results of individual studies | 20 | For all outcomes considered (benefits or harms), present, for each study: (1) simple summary data for each intervention group, and (2) effect estimates and confidence intervals. Modified approaches may be needed to deal with information from larger networks. | Table 1 |
| Synthesis of results | 21 | Present results of each meta-analysis done, including confidence/credible intervals. In larger networks, authors may focus on comparisons versus a particular comparator (e.g. placebo or standard care), with full findings presented in an appendix. League tables and forest plots may be considered to summarize pairwise comparisons. If additional summary measures were explored (such as treatment rankings), these should also be presented. | 3.3 3.4 Outcomes  Figure 3a, Figure 3b, Figure 4, Figure S2a, Figure S2b, Figure S3, Table 2a, Table 2b. |
| Exploration for inconsistency | S5 | Describe results from investigations of inconsistency. This may include such information as measures of model fit to compare consistency and inconsistency models, P values from statistical tests, or summary of inconsistency estimates from different parts of the treatment network. | 3.5 Inconsistency test  Table 5a, Table 5b, Table 6 |
| Risk of bias across | 22 | Present results of any assessment of risk of bias across studies for the evidence base being studied. | 3.7 Publication bias, Figure S5 |
| Additional analyses | 23 | Give results of additional analyses, if done (e.g., sensitivity or subgroup analyses, meta-regression analyses, alternative network geometries studied, alternative choice of prior distributions for Bayesian analyses, and so forth). | 3.6 Sensitivity analysis  Figure S4a, Figure S4b |
| **Discussion** |  |  |  |
| Summary of evidence | 24 | Summarize the main findings, including the strength of evidence for each main outcome; consider their relevance to key groups. | Discussion  4.1 Findings and implications |
| Limitations | 25 | Discuss limitations at study and outcome level (e.g., risk of bias), and at review level (e.g., incomplete retrieval of identified research, reporting bias). Comment on the validity of the assumptions, such as transitivity and consistency. Comment on any concerns regarding network geometry (e.g., avoidance of certain comparisons). | Discussion  4.4 Limitations |
| Conclusions | 26 | Provide a general interpretation of the results in the context of other evidence, and implications for future research. | 5 Conclusion |
| **Funding** |  |  |  |
| Funding | 27 | Describe sources of funding for the systematic review and other support (e.g., supply of data); role of funders for the systematic review. This should also include information regarding whether funding has been received from manufacturers of treatments in the network and/or whether some of the authors are content experts with professional conflicts of interest that could affect use of treatments in the network. | Funding |

PICOS, population, intervention, comparators, outcomes, study design.

**Table S2 -** Keywords and search results in different databases

| Database | Keyword | Filter | Date | Results |
| --- | --- | --- | --- | --- |
| PubMed | ('primary dysmenorrhea' OR 'dysmenorrhea' OR 'menstrual cramps' OR 'painful periods') AND ('exercise' OR 'yoga' OR 'aerobic' OR 'training' OR 'sports' OR 'physical activity' OR 'workout' OR 'fitness' OR 'training') AND ('randomized' OR 'randomised' OR 'random') | NA | February 2, 2024 | 182 |
| Cochrane Reviews | ('primary dysmenorrhea' OR 'dysmenorrhea' OR 'menstrual cramps' OR 'painful periods') AND ('exercise' OR 'yoga' OR 'aerobic' OR 'training' OR 'sports' OR 'physical activity' OR 'workout' OR 'fitness' OR 'training') AND ('randomized' OR 'randomised' OR 'random') | Title Abstract  Keyword | February 2, 2024 | 99 |
| Cochrane CENTRAL | ('primary dysmenorrhea' OR 'dysmenorrhea' OR 'menstrual cramps' OR 'painful periods') AND ('exercise' OR 'yoga' OR 'aerobic' OR 'training' OR 'sports' OR 'physical activity' OR 'workout' OR 'fitness' OR 'training') AND ('randomized' OR 'randomised' OR 'random') | Title Abstract  Keyword | February 2, 2024 | 6771 |
| Web of Science | ('primary dysmenorrhea' OR 'dysmenorrhea' OR 'menstrual cramps' OR 'painful periods') AND ('exercise' OR 'yoga' OR 'aerobic' OR 'training' OR 'sports' OR 'physical activity' OR 'workout' OR 'fitness' OR 'training') AND ('randomized' OR 'randomised' OR 'random') | NA | February 2, 2024 | 189 |
| ClinicalTrials.gov | ('primary dysmenorrhea' OR 'dysmenorrhea' OR 'menstrual cramps' OR 'painful periods') AND ('exercise' OR 'yoga' OR 'aerobic' OR 'training' OR 'sports' OR 'physical activity' OR 'workout' OR 'fitness' OR 'training') AND ('randomized' OR 'randomised' OR 'random') | Condition  or disease | February 2, 2024 | 12 |

NA: not applied

**Table S3** - Excluded studies and reasons

| Citations | Reasons |
| --- | --- |
| Monori A, Csakvari T, Karacsony I, Ferenczy M, Fusz K, Olah A, et al. Comparing the effect of progressive relaxation and perineal strengthening interval exercises among women with primary dysmenorrhoea to reduce menstrual cramps. Value in health. 2017;20(9):A519. | No 4‑ or 8-week time points: 5-week study |
| Günebakan Ö, Acar M. The effect of tele-yoga training in healthy women on menstrual symptoms, quality of life, anxiety-depression level, body awareness, and self-esteem during covid-19 pandemic. Ir J Med Sci. 2023;192(1):467-79. | No 4‑ or 8-week time points: 6-week study |
| Huang WC, Chiu PC, Ho CH. The sprint-interval exercise using a spinning bike improves physical fitness and ameliorates primary dysmenorrhea symptoms through hormone and inflammation modulations: a randomized controlled trial. J Sports Sci Med. 2022;21(4):595-607. | No 4‑ or 8-week time points: 10-week study |
| Aksu A, Vefikuluçay Yılmaz D. The effect of yoga practice on pain intensity, menstruation symptoms and quality of life of nursing students with primary dysmenorrhea. Health Care Women Int. 2024:1-15. | No 4- or 8-week time points: 12-week study |
| Fathy FT, Gonied AS, El-Dosoky MM, Mohamed SL, Mohamed NS. Effect of aerobic exercises on intensity of primary dysmenorrhea among nursing students. NeuroQuantology. 2022;20(10):6639‐54. | No 4‑ or 8-week time points: 12-week study |
| Nag U, Kodali M. Meditation and yoga as alternative therapy for primary dysmenorrhea. International Journal of Medical and Pharmaceutical Sciences. 2013;3:39-44. | No 4‑ or 8-week time points: 12-week study |
| Arora A, Yardi S, Gopal S. Effect of 12-weeks of aerobic exercise on primary dysmenorrhea. Indian Journal of Physiotherapy and Occupational Therapy - An International Journal. 2014;8:130. | No 4‑ or 8-week time points: 12-week study |
| Yilmaz-Akyuz E, Aydin-Kartal Y. The effect of diet and aerobic exercise on premenstrual syndrome: randomized controlled trial. Revista De Nutricao-Brazilian Journal of Nutrition. 2019;32:e180246. | No 4‑ or 8-week time points: 12-week study |
| Yonglitthipagon P, Muansiangsai S, Wongkhumngern W, Donpunha W, Chanavirut R, Siritaratiwat W, et al. Effect of yoga on the menstrual pain, physical fitness, and quality of life of young women with primary dysmenorrhea. J Bodyw Mov Ther. 2017;21(4):840-6. | No 4‑ or 8-week time points: 12-week study |
| Yang NY, Kim SD. Effects of a yoga program on menstrual cramps and menstrual distress in undergraduate students with primary dysmenorrhea: a single-blind, randomized controlled trial. J Altern Complement Med. 2016;22(9):732-8. | No 4‑ or 8-week time points: 12-week study |
| Dauneria S, Keswani J. A study on the effect of yoga and naturopathy on dysmenorrhea. Int J Yoga Allied Sci 2014;3(1):38-42. | No 4‑ or 8-week time points: 12-week study |
| Paithankar DSM, Hande DN. Effectiveness of pilates over conventional physiotherapeutic treatment in females with primary dysmenorrhea. IOSR Journal of Dental and Medical Sciences. 2016;15(4):156-63. | No 4‑ or 8-week time points: 12-week study |
| Thoke A, Gawali M. Effects of surya namaskar and yoga nidra on physical problems of adolescent girls during their menstruation. GOEIJ 2015:4. | No 4‑ or 8-week time points: 24-week study |
| Gupta R, Kaur S, Kaur A. Comparison to assess the effectiveness of active exercises and dietary ginger vs. active exercises on primary dysmenorrheal among adolescent girls. Nurs Midwifery Res. 2013;9:168‑77. | Comparing a non-exercise intervention only: dietary ginger |
| Shirvani MA, Motahari-Tabari N, Alipour A. Use of ginger versus stretching exercises for the treatment of primary dysmenorrhea: a randomized controlled trial. J Integr Med. 2017;15(4):295-301. | Comparing a non-exercise intervention only: ginger capsules |
| Chaudhuri A, Singh A, Dhaliwal L. A randomised controlled trial of exercise and hot water bottle in the management of dysmenorrhoea in school girls of chandigarh, india. Indian J Physiol Pharmacol. 2013;57(2):114-22. | Comparing a non-exercise intervention only: hot water bottle |
| Motahari-Tabari N, Shirvani MA, Alipour A. Comparison of the effect of stretching exercises and mefenamic acid on the reduction of pain and menstruation characteristics in primary dysmenorrhea: A randomized clinical trial. Oman Med J. 2017;32(1):47-53. | Comparing a non-exercise intervention only: mefenamic acid |
| Kanwal R, Masood T, Awan WA, Babur M, Baig MS. Effectiveness of TENS versus stretching exercises on primary dysmenorrhea in students. International Journal of Rehabilitation Sciences. 2017;5(2):18-24. | Comparing a non-exercise intervention only: transcutaneous electrical nerve stimulation (TENS) |
| El-Bably EM, Abd El-Aziz KS, El-Bandrawy AM, Abo El-Enein MF. Effect of pilates exercise on primary dysmenorrhea. The Medical Journal of Cairo University. 2019:1187-92. | Comparing a non-exercise intervention only: transcutaneous electrical nerve stimulation (TENS) |
| Imtiaz I, Riaz H. Effects of high intensity aerobic training on symptomatology of primary dysmenorrhoea. Journal of the Pakistan Medical Association. 2022;72(12):2515‐8. | No effective paired comparison could be included in our network meta-analysis model after combing two aerobic trainings with different protocol |
| Sarhadi S, Ramezani A, Gholami M, Taheri H. The effect of an aerobic training cycle in the morning and evening on primary dysmenorrhea and some physiological variables in matured girls. Iranian journal of obstetrics, gynecology and infertility. 2015;18(162):11‐20. | No effective paired comparison could be included in our network meta-analysis model after combing two aerobic trainings with different protocol |
| Shavandi N, Taghian F, Soltani V. The effect of isometric exercise on primary dismenorrhea. Journal of Arak University of Medical Sciences. 2010;13(1):71-7. | No data available. The means and standard deviations are not provided in the full text. |
| Lorzadeh N, Kazemirad Y, Kazemirad N. The effect of corrective and therapeutic exercises on bleeding volume and severe menstrual pain in non-athletic women. J Obstet Gynaecol. 2021;41(7):1121-6. | No data available. The means and standard deviations are not provided in the full text. |
| Mahvash N, Alijani E, Kohandel M, H S. The effect of physical activity on primary dysmenorrhea of female university students. World Appl Scs J 2012;17:1246-52. | No data available. The means and standard deviations are not provided in the full text. |
| Israel RG, Sutton M, O'Brien KF. Effects of aerobic training on primary dysmenorrhea symptomatology in college females. J Am Coll Health. 1985;33(6):241-4. | Insufficient data. The standard deviations are not provided in the full text. |
| Gokulakrishnan J, Momin RS. Effectiveness of Kegel's exercises on reducing pain and menstrual cramps in dysmenorrhea among college hostel girls. International Journal of Research Publication and Reviews. 2022;3(8):340-3. | Insufficient data. The standard deviations are not provided in the full text. |
| Sutar A, Paldhikar S, Shikalgar N, Ghodey S. Effect of aerobic exercises on primary dysmenorrhoea in college students. IOSR Journal of Nursing and Health Science. 2016;05:20-4. | Insufficient data. The results of control group are not provided in the full text. |
| Aboushady RM-N, El-saidy TMK. Effect of home-based stretching exercises and menstrual care on primary dysmenorrhea and premenstrual symptoms among adolescent girls. IOSR Journal of Nursing and Health Science. 2016(5):10-7. | Insufficient data. The results of control group are not provided in the full text. |
| Cholbeigi E, Rezaienik S, Safari N, Lissack K, Griffiths MD, Alimoradi Z. Are health promoting lifestyles associated with pain intensity and menstrual distress among iranian adolescent girls? BMC Pediatr. 2022;22(1):574. | Not a randomized-controlled trial |
| Kovács Z, Hegyi G, Szőke H. The Effect of Exercise on Pulsatility Index of Uterine Arteries and Pain in Primary Dysmenorrhea. J Clin Med. 2023;12(22):7021. | Not a randomized-controlled trial |
| Golub LJ, Menduke H, Lang WR. Exercise and dysmenorrhea in young teenagers: a 3-year study. Obstet Gynecol. 1968;32(4):508-11. | Not a randomized-controlled trial |
| Hubbell JW. Specific and non-specific exercise for the relief of dysmenorrhea. Res Q. 1949;20(4):378-86. | Not a randomized-controlled trial |
| Lundquist C. Use of the billig exercise for dysmenorrhea for college women. Res Q. 1947;18(1):45-53. | Not a randomized-controlled trial |
| Kannan P, Claydon LS, Miller D, Chapple CM. Vigorous exercises in the management of primary dysmenorrhea: a feasibility study. Disabil Rehabil. 2015;37(15):1334-9. | Not a randomized-controlled trial |
| Mirbagher-Ajorpaz N, Adib-Hajbaghery M, Mosaebi F. The effects of acupressure on primary dysmenorrhea: A randomized controlled trial. Complement Ther Clin Pract. 2011;17(1):33-6. | Non-exercise intervention: acupressure |
| Özgül S, Üzelpasaci E, Orhan C, Baran E, Beksaç MS, Akbayrak T. Short-term effects of connective tissue manipulation in women with primary dysmenorrhea: a randomized controlled trial. Complementary Therapies in Clinical Practice. 2018;33:1-6. | Non-exercise intervention: connective tissue manipulation, which was performed by a physical therapist, not by the participants themselves |
| Chantler I, Mitchell D, Fuller A. Diclofenac potassium attenuates dysmenorrhea and restores exercise performance in women with primary dysmenorrhea. J Pain. 2009;10(2):191-200. | Non-exercise intervention: diclofenac |
| Gaubeca-Gilarranz A, Fernández-de-Las-Peñas C, Medina-Torres JR, Seoane-Ruiz JM, Company-Palonés A, Cleland JA, et al. Effectiveness of dry needling of rectus abdominis trigger points for the treatment of primary dysmenorrhoea: a randomised parallel-group trial. Acupunct Med. 2018;36(5):302-10. | Non-exercise intervention: dry needling |
| Keshavarzi F, Mahmoudzadeh F, Brand S, Sadeghi Bahmani D, Akbari F, Khazaie H, et al. Both melatonin and meloxicam improved sleep and pain in females with primary dysmenorrhea-results from a double-blind cross-over intervention pilot study. Arch Womens Ment Health. 2018;21(6):601-9. | Non-exercise intervention: melatonin and meloxicam |
| Vaziri F, Hoseini A, Kamali F, Abdali K, Hadianfard M, Sayadi M. Comparing the effects of aerobic and stretching exercises on the intensity of primary dysmenorrhea in the students of universities of bushehr. J Family Reprod Health. 2015;9(1):23-8. | Not using visual analogue scale (VAS) for pain intensity measurement |
| Zainab S, Nithyashree P, Jumanah R, Kamalakannan M, Suganthirababu P, Kumaresan A. A study to compare the effectiveness of core strengthening exercises for phase I and phase II of menstrual cycle in primary dysmenorrhea subjects. Biomedicine (india). 2021;41(2):315‐7. | Not using visual analogue scale (VAS) for pain intensity measurement |
| Nag U, Dip P, Kodali M. Effect of yoga on primary dysmenorrhea and stress in medical students. IOSR Journal of Dental and Medical Sciences. 2013;4:69-73. | Not using visual analogue scale (VAS) for pain intensity measurement |
| Behbahani BM, Ansaripour L, Akbarzadeh M, Zare N, Hadianfard MJ. Comparison of the effects of acupressure and self-care behaviors training on the intensity of primary dysmenorrhea based on mcgill pain questionnaire among shiraz university students. J Res Med Sci. 2016;21:104. | Not using visual analogue scale (VAS) for pain intensity measurement |
| Kannan P, Chapple CM, Miller D, Claydon LS, Baxter GD. Menstrual pain and quality of life in women with primary dysmenorrhea: Rationale, design, and interventions of a randomized controlled trial of effects of a treadmill-based exercise intervention. Contemp Clin Trials. 2015;42:81-9. | Protocol publication: no results could be retrieved |
| Kannan P, Cheung KK, Lau BW, Li L, Chen H, Sun F. A mixed-methods study to evaluate the effectiveness and cost-effectiveness of aerobic exercise for primary dysmenorrhea: A study protocol. PLoS One. 2021;16(8):e0256263. | Protocol publication: no results could be retrieved |
| Maged AM, Abbassy AH, Sakr HRS, Elsawah H, Wagih H, Ogila AI, et al. Effect of swimming exercise on premenstrual syndrome. Arch Gynecol Obstet. 2018;297(4):951-9. | Not measuring pain intensity |
| Rani M, Singh U, Agrawal GG, Natu SM, Kala S, Ghildiyal A, et al. Impact of yoga nidra on menstrual abnormalities in females of reproductive age. J Altern Complement Med. 2013;19(12):925-9. | Not measuring pain intensity |
| Sandhiya M, Senthil Selvam P, Manoj Abraham M, Palekar TJ, Sundaram MS, Kumari P, et al. A study to compare the effects of aerobic exercise versus core strengthening exercise among college girls with primary dysmenorrhea. International journal of research in pharmaceutical sciences. 2020;11(Special Issue 4):2692‐7. | Violating transitivity assumption: only including underweight female (BMI < 19) |
| Azima S, Rajaei Bakhshayesh H, Abbasnia K, Kaviani M, Sayadi M. The effect of isometric exercises on primary dysmenorrhea: a randomized controlled clinical trial. Galen Medical Journal. 2015;4(1):26-32. | Overlapping population. The author conducted a 3-arm study that was already included in our network meta-analysis. This publication is using only 2 of the arms from the original study. |

**Table S4** - Detailed quality assessment of included studies using Cochrane risk of bias 2 tool

| First author & Year | Randomization  process | Intervention  adherence | Missing  outcome data | Outcome  measurement | Selective  reporting | Overall  RoB |
| --- | --- | --- | --- | --- | --- | --- |
| Abbaspour 2004 | S^1^ | L | L | L | L | S |
| Rakhshaee 2011 | S^1^ | L | L | L | L | S |
| Sakuma 2012 | L | L | L | L | L | L |
| Salehi 2012 | S^1^ | L | L | L | L | S |
| Shahr-jerdy 2012 | S^1^ | L | L | L | L | S |
| Kaur 2013 | S^1^ | L | L | L | L | S |
| Reyhani 2013 | S^1^ | L | L | L | L | S |
| Rezvani 2013 | S^1^ | L | L | L | L | S |
| Siahpour 2013 | S^1^ | L | L | L | L | S |
| Gamit 2014 | S^1^ | L | L | L | L | S |
| Azima 2015 | L | L | L | L | L | L |
| Ortiz 2015 | L | L | L | L | L | L |
| Patel 2015 | S^1^ | L | L | L | H^3^ | H |
| Heidarianpour 2016 | S^1^ | L | L | L | L | S |
| Nasri 2016 | S^1^ | L | L | L | L | S |
| Saleh 2016 | L | L | L | L | L | L |
| Shah 2016 | S^1^ | L | L | L | L | S |
| Tharani 2018 | S^1^ | L | L | L | L | S |
| Akbas 2019 | S^1^ | L | L | L | L | S |
| Chen 2019 | L | L | L | L | L | L |
| Heidarimoghadam 2019 | S^1^ | L | L | L | L | S |
| Kannan 2019 | L | L | L | L | L | L |
| Samy 2019 | L | L | L | L | L | L |
| Kirmizigil 2020 | L | L | L | L | L | L |
| Çelik 2021 | S^2^ | L | L | L | L | S |
| Kirca 2021 | L | L | L | L | L | L |
| Ozturk 2022 | S^2^ | L | L | L | L | S |
| Yildiz 2022 | L | L | L | L | L | L |
| Yosri 2022 | L | L | L | L | L | L |

^1^ Randomization details were not provided.

^2^ The allocation sequence was predictable.

^3^ The study collected data on pain duration, but did not report it.

H, high risk of bias; L, low risk of bias; S, some risk of bias.

**Table S5** - Inconsistency test results of the mean difference in menstrual pain reduction after exercise

| **At four weeks** | **Studies** | **NMA** | **Direct** | **Indirect** | **Difference** | **95CIL** | **95CIU** | ***p* value** |
| --- | --- | --- | --- | --- | --- | --- | --- | --- |
| Aerobic : Control | 5 | -1.83 | -1.83 | - | - | - | - | - |
| Aerobic : Kegel | 0 | -0.76 | - | -0.76 | - | - | - | - |
| Aerobic : Mixed | 0 | -0.56 | - | -0.56 | - | - | - | - |
| Aerobic : Relaxation | 0 | 1.72 | - | 1.72 | - | - | - | - |
| Aerobic : Strength | 0 | 0.46 | - | 0.46 | - | - | - | - |
| Aerobic : Yoga | 0 | -0.21 | - | -0.21 | - | - | - | - |
| Kegel : Control | 1 | -1.07 | -1.07 | - | - | - | - | - |
| Mixed : Control | 2 | -1.27 | -0.90 | -2.23 | 1.33 | -2.81 | 5.47 | 0.53 |
| Relaxation : Control | 4 | -3.56 | -3.52 | -3.93 | 0.42 | -4.71 | 5.54 | 0.87 |
| Strength : Control | 6 | -2.29 | -2.04 | -7.68 | 5.64 | -0.31 | 11.59 | 0.06 |
| Yoga : Control | 3 | -1.63 | -1.87 | -0.54 | -1.33 | -5.47 | 2.81 | 0.53 |
| Kegel : Mixed | 0 | 0.20 | - | 0.20 | - | - | - | - |
| Kegel : Relaxation | 0 | 2.49 | - | 2.49 | - | - | - | - |
| Kegel : Strength | 0 | 1.22 | - | 1.22 | - | - | - | - |
| Kegel : Yoga | 0 | 0.56 | - | 0.56 | - | - | - | - |
| Mixed : Relaxation | 0 | 2.29 | - | 2.29 | - | - | - | - |
| Mixed : Strength | 0 | 1.02 | - | 1.02 | - | - | - | - |
| Mixed : Yoga | 1 | 0.36 | -0.36 | 0.97 | -1.33 | -5.47 | 2.81 | 0.53 |
| Relaxation : Strength | 2 | -1.26 | -0.57 | -2.24 | 1.67 | -1.74 | 5.08 | 0.34 |
| Relaxation : Yoga | 0 | -1.93 | - | -1.93 | - | - | - | - |
| Strength : Yoga | 0 | -0.67 | - | -0.67 | - | - | - | - |
| **At eight weeks** | **Studies** | **NMA** | **Direct** | **Indirect** | **Difference** | **95CIL** | **95CIU** | ***p* value** |
| Aerobic : Control | 7 | -2.77 | -2.50 | -4.33 | 1.83 | -0.58 | 4.25 | 0.14 |
| Aerobic : Kegel | 1 | 0.15 | 0.54 | -0.23 | 0.77 | -2.79 | 4.33 | 0.67 |
| Aerobic : Mixed | 0 | 0.90 | - | 0.90 | - | - | - | - |
| Aerobic : Relaxation | 0 | 1.10 | - | 1.10 | - | - | - | - |
| Aerobic : Strength | 1 | 0.40 | -1.20 | 0.85 | -2.05 | -4.83 | 0.73 | 0.15 |
| Aerobic : Yoga | 1 | -0.02 | -0.65 | 0.28 | -0.93 | -3.99 | 2.13 | 0.55 |
| Kegel : Control | 2 | -2.93 | -2.68 | -4.57 | 1.89 | -3.13 | 6.91 | 0.46 |
| Mixed : Control | 2 | -3.67 | -3.68 | -3.66 | -0.02 | -3.32 | 3.28 | 0.99 |
| Relaxation : Control | 2 | -3.87 | -3.67 | -5.29 | 1.62 | -3.40 | 6.63 | 0.53 |
| Strength : Control | 7 | -3.17 | -3.42 | -1.50 | -1.91 | -4.52 | 0.70 | 0.15 |
| Yoga : Control | 3 | -2.75 | -2.88 | -2.36 | -0.52 | -3.44 | 2.40 | 0.73 |
| Kegel : Mixed | 0 | 0.75 | - | 0.75 | - | - | - | - |
| Kegel : Relaxation | 0 | 0.94 | - | 0.94 | - | - | - | - |
| Kegel : Strength | 0 | 0.25 | - | 0.25 | - | - | - | - |
| Kegel : Yoga | 0 | -0.18 | - | -0.18 | - | - | - | - |
| Mixed : Relaxation | 0 | 0.20 | - | 0.20 | - | - | - | - |
| Mixed : Strength | 0 | -0.50 | - | -0.50 | - | - | - | - |
| Mixed : Yoga | 1 | -0.92 | -0.91 | -0.93 | 0.02 | -3.28 | 3.32 | 0.99 |
| Relaxation : Strength | 1 | -0.69 | -1.20 | -0.22 | -0.98 | -4.47 | 2.51 | 0.58 |
| Relaxation : Yoga | 0 | -1.12 | - | -1.12 | - | - | - | - |
| Strength : Yoga | 0 | -0.42 | - | -0.42 | - | - | - | - |

95CIL: lower limit of 95% confidence interval; 95CIU: upper limit of 95% confidence interval;

NMA: network meta-analysis

**Table S6** - Inconsistency test results for difference in risk of dropout at eight weeks

| Comparison | Studies | NMA | Direct | Indirect | Difference | 95CIL | 95CIU | *p* value |
| --- | --- | --- | --- | --- | --- | --- | --- | --- |
| Aerobic : Control | 7 | 0.00 | 0.00 | -0.03 | 0.03 | -0.07 | 0.13 | 0.57 |
| Aerobic : Kegel | 1 | 0.00 | 0.00 | 0.00 | 0.00 | -0.18 | 0.18 | 0.98 |
| Aerobic : Mixed | 0 | 0.03 | - | 0.03 | - | - | - | - |
| Aerobic : Relaxation | 0 | 0.11 | - | 0.11 | - | - | - | - |
| Aerobic : Strength | 1 | 0.00 | 0.00 | 0.00 | 0.00 | -0.12 | 0.12 | 0.99 |
| Aerobic : Yoga | 1 | 0.04 | 0.00 | 0.05 | -0.05 | -0.17 | 0.06 | 0.35 |
| Kegel : Control | 2 | 0.00 | 0.00 | 0.00 | 0.00 | -0.23 | 0.24 | 0.98 |
| Mixed : Control | 2 | -0.03 | -0.03 | -0.04 | 0.01 | -0.10 | 0.12 | 0.86 |
| Relaxation : Control | 2 | -0.11 | -0.08 | -0.28 | 0.21 | -0.05 | 0.46 | 0.11 |
| Strength : Control | 7 | 0.00 | 0.00 | 0.02 | -0.02 | -0.14 | 0.10 | 0.73 |
| Yoga : Control | 3 | -0.04 | -0.05 | -0.02 | -0.02 | -0.13 | 0.08 | 0.66 |
| Kegel : Mixed | 0 | 0.03 | - | 0.03 | - | - | - | - |
| Kegel : Relaxation | 0 | 0.11 | - | 0.11 | - | - | - | - |
| Kegel : Strength | 0 | 0.00 | - | 0.00 | - | - | - | - |
| Kegel : Yoga | 0 | 0.04 | - | 0.04 | - | - | - | - |
| Mixed : Relaxation | 0 | 0.07 | - | 0.07 | - | - | - | - |
| Mixed : Strength | 0 | -0.03 | - | -0.03 | - | - | - | - |
| Mixed : Yoga | 1 | 0.00 | 0.00 | 0.01 | -0.01 | -0.12 | 0.10 | 0.86 |
| Relaxation : Strength | 1 | -0.11 | -0.24 | -0.05 | -0.18 | -0.39 | 0.02 | 0.08 |
| Relaxation : Yoga | 0 | -0.07 | - | -0.07 | - | - | - | - |
| Strength : Yoga | 0 | 0.04 | - | 0.04 | - | - | - | - |

95CIL: lower limit of 95% confidence interval; 95CIU: upper limit of 95% confidence interval;

NMA: network meta-analysis
